# Education and myopia: assessing the direction of causality by mendelian randomisation

**DOI:** 10.1136/bmj.k2022

**Published:** 2018-06-06

**Authors:** Edward Mountjoy, Neil M Davies, Denis Plotnikov, George Davey Smith, Santiago Rodriguez, Cathy E Williams, Jeremy A Guggenheim, Denize Atan

**Affiliations:** 1 MRC Integrative Epidemiology Unit, Bristol Medical School, University of Bristol, Bristol, UK; 2 Population Health Sciences, Bristol Medical School, University of Bristol, Bristol, UK; 3 School of Optometry and Vision Sciences, Cardiff University, Cardiff, UK; 4 Translational Health Sciences, Bristol Medical School, University of Bristol, Biomedical Sciences Building, Bristol BS8 1TD, UK

## Abstract

**Objectives:**

To determine whether more years spent in education is a causal risk factor for myopia, or whether myopia is a causal risk factor for more years in education.

**Design:**

Bidirectional, two sample mendelian randomisation study.

**Setting:**

Publically available genetic data from two consortiums applied to a large, independent population cohort. Genetic variants used as proxies for myopia and years of education were derived from two large genome wide association studies: 23andMe and Social Science Genetic Association Consortium (SSGAC), respectively.

**Participants:**

67 798 men and women from England, Scotland, and Wales in the UK Biobank cohort with available information for years of completed education and refractive error.

**Main outcome measures:**

Mendelian randomisation analyses were performed in two directions: the first exposure was the genetic predisposition to myopia, measured with 44 genetic variants strongly associated with myopia in 23andMe, and the outcome was years in education; and the second exposure was the genetic predisposition to higher levels of education, measured with 69 genetic variants from SSGAC, and the outcome was refractive error.

**Results:**

Conventional regression analyses of the observational data suggested that every additional year of education was associated with a more myopic refractive error of −0.18 dioptres/y (95% confidence interval −0.19 to −0.17; P<2e-16). Mendelian randomisation analyses suggested the true causal effect was even stronger: −0.27 dioptres/y (−0.37 to −0.17; P=4e-8). By contrast, there was little evidence to suggest myopia affected education (years in education per dioptre of refractive error −0.008 y/dioptre, 95% confidence interval −0.041 to 0.025, P=0.6). Thus, the cumulative effect of more years in education on refractive error means that a university graduate from the United Kingdom with 17 years of education would, on average, be at least −1 dioptre more myopic than someone who left school at age 16 (with 12 years of education). Myopia of this magnitude would be sufficient to necessitate the use of glasses for driving. Sensitivity analyses showed minimal evidence for genetic confounding that could have biased the causal effect estimates.

**Conclusions:**

This study shows that exposure to more years in education contributes to the rising prevalence of myopia. Increasing the length of time spent in education may inadvertently increase the prevalence of myopia and potential future visual disability.

## Introduction

Myopia, or short-sightedness, is one of the leading causes of visual disability worldwide, and its prevalence is increasing rapidly.^1^ ^2^ Myopia is a refractive defect of the eye that causes light to focus in front of, rather than on, the retina, usually because the axial length of the eye is too long. As a result, distant objects appear blurred and close objects appear clearly (short-sight). The symptoms of myopia can be alleviated with spectacles, contact lenses, or refractive surgery, but irrespective of visual correction, the risk of complications from potentially blinding conditions such as retinal detachment, glaucoma, and myopic maculopathy, increase with the longer axial lengths associated with high myopia.^3^ ^4^ ^5^ Currently, 30-50% of adults in the United States and Europe are myopic, with levels of 80-90% reported in school leavers aged 17 or 18 years in Singapore, South Korea, China, and other high income Eastern and South East Asian countries,^1^ ^2^ ^5^ ^6^ ^7^ ^8^ where myopic maculopathy has become one the most common causes of untreatable blindness.^7^ Based on existing trends, the number of people affected by myopia worldwide is expected to increase from 1.4 billion to 5 billion by 2050, affecting about 50% of the world’s population.^7^ Almost 10% of these people (around 9 million) will have high myopia.^7^

For more than a century, myopia has been associated with higher levels of educational attainment,^9^ ^10^ but despite evidence from observational studies for an association between myopia and years of schooling or educational attainment, causal evidence for a role of education on myopia is lacking.^6^ ^11^ ^12^ ^13^ Both myopia and educational attainment have a heritable component^14^ ^15^ ^16^ ^17^ ^18^ ^19^ ^20^; however, genetics cannot explain the rapid increase in the prevalence of myopia over one or two generations. The current increased prevalence of myopia, particularly high myopia, seems to be linked to an increasingly earlier age of onset and higher rate of progression in childhood,^21^ ^22^ since the condition tends to remain relatively stable during adulthood (until myopic shifts occur secondary to the development of cataracts).^23^ Randomised controlled trials have shown convincingly that time spent outdoors in childhood partially protects against the development of myopia,^24^ ^25^ but the association between myopia and time spent by children doing near work activities, such as reading, is less consistent across studies.^11^ ^26^ Furthermore, the time children spend outdoors is typically independent of their near work activities, as measures of the two are generally uncorrelated.^27^ ^28^ ^29^ Consequently, it is not known with any certainty whether more years in education causes myopia, children with myopia spend more time on near work leading to better educational outcomes, children with myopia are more intelligent, or, indeed, an association with another confounding factor, such as socioeconomic position, leads to more years in education and myopia,^6^ ^11^ ^12^ ^13^ ^30^ since randomised trials that limit education in children would be unethical.

Mendelian randomisation is a type of instrumental variable analysis^31^ that uses genetic variants associated with a risk factor (eg, education) as proxies for an environmental exposure to make causal inferences about the effect of the exposure on the outcome of interest (eg, myopia). This approach reduces bias from confounding and reverse causation, to which observational epidemiology studies are susceptible. It exploits the fact that genotypes are randomly assigned at conception. Hence, mendelian randomisation has been likened to a randomised trial by genotype, since genetic variants are not modifiable and are largely free from confounding.^32^ ^33^ With the recent availability of data from two large scale genome-wide association studies (GWAS) for educational attainment^34^ and myopia,^15^ together with the genotypes of approximately 488 000 participants in the UK Biobank, an investigation of the causal relation between years in education and myopia by bidirectional mendelian randomisation analyses^35^ became possible with unprecedented statistical power. We investigated whether more time spent in education is a causal risk factor for myopia.

## Methods

### Study cohorts

*23andMe*—Pickrell et al^15^ reported the results of a GWAS for self reported myopia in a sample of 191 843 people of European descent (106 086 cases, 85 757 controls) carried out by the personal genomics company 23andMe (CA, USA). Myopia was ascertained by the questionnaire item “Have you ever been diagnosed by a doctor with nearsightedness (near objects are clear, far objects are blurry)?”

*Social Science Genetic Association Consortium (SSGAC)*—Okbay et al^34^ reported the results of a large meta-analysis of GWAS for educational attainment in 293 723 people of European descent. Educational attainment was defined by whether the participant attained a given level of schooling, based on the International Standard Classification of Education (1997) scale.^36^

*UK Biobank*—Cross sectional data from the baseline assessment of the UK Biobank project was collected between 2006 and 2010.^37^ UK Biobank recruited 502 664 participants aged 40 to 69 years through 22 assessment centres across the UK. One of two platforms was used to determine the genotype of participants: the BiLEVE Axiom array (Affymetrix, High Wycombe, UK) or the Biobank Axiom array (Affymetrix). All participants completed sociodemographic questionnaires, which included questions on past educational and professional qualifications. In the latter stages of recruitment, an ophthalmic assessment was introduced, and this was completed by approximately 23% of participants.

### Definitions

*Education—*We determined the time spent in education by questionnaire as defined by the question for age of completed full time education in UK Biobank (n=336 826 participants completed the questionnaire at the baseline visit). The question was ascertained only for participants who did not have a college or university degree. To harmonise the educational outcome measure in UK Biobank (time spent in education) with the number of years spent in schooling (EduYears) variable in the SSGAC study,^34^ we coded participants with a college or university degree as having left full time education at age 21 years. Similarly, we assigned a value of 15 years for those participants who reported being aged less than 15 on completion of full time education. As schooling systems differ between countries, we included in the analyses only participants born in England, Scotland, or Wales

*Refractive error*—Measures of visual function were not performed from the start of recruitment for UK Biobank. Consequently, only a subset of participants underwent measurements of refractive error (n=127 412). This was measured by non-cycloplegic autorefraction (RC5000 autorefractor; Tomey, Phoenix, AZ) after removal of spectacles or contact lenses. Although cycloplegic eye drops were not used (ie, the effect of accommodation on measurements of refractive error was not controlled), only adults in whom the effects of accommodation would be minimal were recruited to UK Biobank.^38^ Up to 10 measurements were taken. If the autorefractor reading was flagged as unreliable then we excluded the measurement. Spherical power and cylindrical power were averaged over repeat measurements. We calculated mean spherical equivalent refractive error for each eye using the formula spherical power+0.5×cylindrical power. We took the average of the left and right mean spherical equivalent as the participant’s refractive error in dioptres and used this value in subsequent analyses (n=127 412). For participants with repeat measurements from separate visits (baseline visit and subsequent visits), we used only the baseline measurement. We excluded from the analyses those with pre-existing eye conditions that could affect refractive error—namely, cataracts, refractive laser eye surgery, injury or trauma resulting in vision loss, or corneal graft surgery. For example, cataracts are associated with a myopic shift in refractive error. We excluded 10 984 individuals with pre-existing eye conditions.

**Fig 1:**
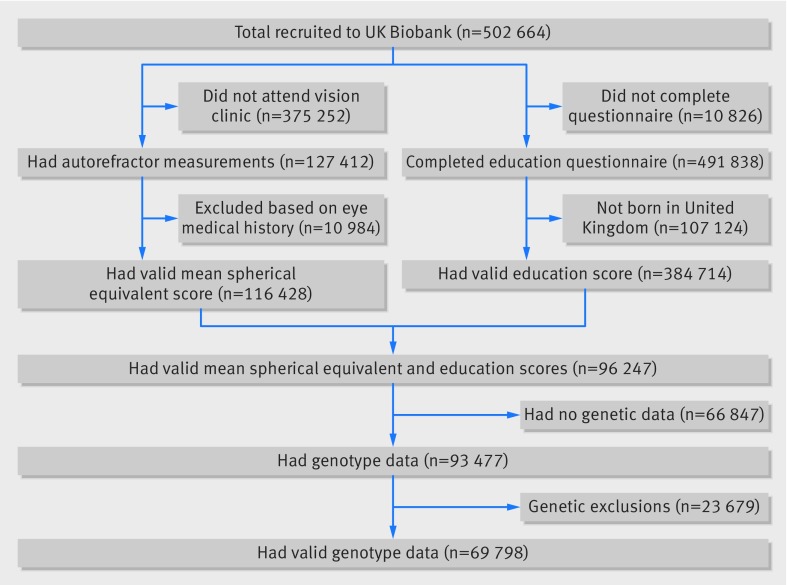
Numbers of participants in UK Biobank who passed validation for mendelian randomisation study. MSE=mean spherical equivalent

In total, 69 798 participants had valid education, refractive error, and genetic data available (fig 1).

### Genotype data

The genetic data in UK Biobank underwent rigorous quality control procedures and was phased and imputed against a reference panel of Haplotype Reference Consortium (HRC), UK10K, and 1000 Genomes Phase 3 haplotypes.^39^ ^40^ Because of a problem with the imputation of UK10K and 1000 Genomes variants, we restricted analyses to HRC variants only. Samples were excluded based on several genotype based criteria: non-European ancestry, relatedness, mismatch between genetic sex and self reported gender, putative aneuploidy, outlying heterozygosity, and excessive missingness.^39^

### Statistical analyses

#### Ordinary least squares observational analyses

Using linear regression adjusted for sex and age in UK Biobank, we assessed observational associations between refractive error and years spent in education. We then repeated the regression with adjustment for additional potentially confounding variables (for example, breast feeding has been reported to be associated with both refractive error^41^ and education^42^): Townsend deprivation index, birth weight, breast fed, and geographical coordinates of place of birth rounded to the nearest kilometre (northing and easting coordinates).

#### Generation of instrumental variables for mendelian randomisation

Pickrell et al^15^ reported the 50 variants most strongly associated with myopia in the study carried out by 23andMe. Six variants (rs5022942, rs10887265, rs71041628, rs34016308, rs11658305, and rs201140091) were not in the HRC panel, leaving 44 for use as genetic instrumental variables in the mendelian randomisation analysis (see supplementary data table).

Okbay et al^34^ used UK Biobank as a replication cohort. Therefore in this study we used only genetic variants and summary statistics from their discovery analysis (http://ssgac.org/documents/EduYears_Discovery_5000.txt). The authors identified 74 variants associated with educational attainment in SSGAC. Five variants (rs9320913, rs148734725, rs544990728, rs114598875, and rs8005528) were not in the HRC panel, leaving 69 variants for use as instrumental variables (see supplementary data table).

For each trait we combined multiple genetic variants into a single weighted allele score. Compared with individual variants, this score has been shown to improve the coverage properties and reduce the bias of instrumental variable estimates.^43^ When constructing allele scores we used effect size estimates from the original GWAS publications to weight variants. To ensure correct coding of the effect allele we harmonised these variants with UK Biobank. We converted the genotype probabilities to effect allele (*a*) and non-effect allele (*A*) dosages. Allele scores were calculated by summing the product of the weights and dosages across all *n* variants (see equations).

**Figure:**
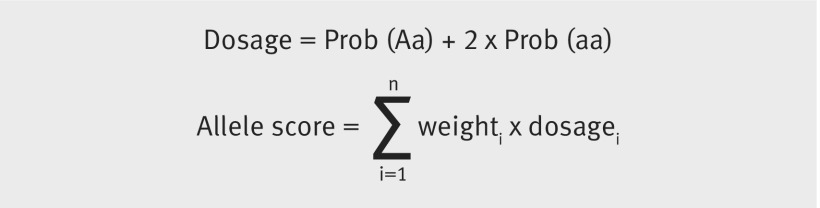


We calculated the proportion of variance in the phenotype variable explained by the allele score instrumental variable, by regressing the phenotype on its respective allele score.

#### Implementation of mendelian randomisation

Mendelian randomisation was implemented using the two stage least squares method in the R package *ivpack*.^44^ We included age and sex as covariates. To assess the risk of weak instrument bias, we used F tests to determine the strength of association in the first stage regressions between allele score and exposure.^45^ Statistical power was assessed using the mRnd online calculator^46^ for a type I error level α=0.05 (http://cnsgenomics.com/shiny/mRnd/).

### Sensitivity analyses

#### Confounding

We used confounding bias plots^47^ ^48^ to assess relative bias in the instrumental variable estimate compared with standard multivariable regression. Such analyses are designed to quantify the bias present in a mendelian randomisation analysis in a manner analogous to examining the effect of adjusting or not adjusting for a potential confounder in a standard regression analysis. Additionally, in supplementary analyses we included suspected confounding factors as covariates (see supplementary table 4). The confounding variables considered^42^ ^49^ ^50^ were the first 10 genetic principal components, Townsend deprivation index, birth weight, breast fed, and place of birth (northing and easting coordinates).

#### Horizontal (genetic) pleiotropy

To investigate the degree of bias in the initial causal estimates due to pleiotropic effects, we used two sensitivity analyses (mendelian randomisation-Egger and weighted median mendelian randomisation). Mendelian randomisation-Egger is not valid for studies in which the instrumental variable-exposure and instrumental variable-outcome associations are calculated in the same sample (as was done for the main analyses in this study). Therefore, we ran the mendelian randomisation-Egger as a split sample analysis, by randomly splitting the sample in half (groups A and B). The supplementary data table shows the associations of the variants and time spent in education and refractive error for each group. Mendelian randomisation-Egger and weighted median methods were implemented using the R package TwoSampleMR (github.com/MRCIEU/TwoSampleMR).^51^

#### Measurement error

To ensure the association between time spent in education and myopia was not an artefact of the non-normal distribution of the variable for age when full time education was completed, we used two alternative methods to recode time spent in education: dichotomisation into age more than 16 years when education was completed and age 16 years or less when education was completed; and excluding those who attended college or university. We compared the results with the original analyses using the continuous variable for age when full time education was completed.

The Durbin-Wu-Hausman test checks for the presence of endogenous variables in a regression model; the presence of such variables leads to biased effect estimates.^52^ ^53^ Effect estimates from the observational analysis and second stage instrumental analysis were tested for endogeneity using the Durbin-Wu-Hausman test.

### Patient involvement

No patients were involved in setting the research question or the outcome measures, nor were they involved in developing plans for recruitment, design, or implementation of the study. No patients were asked to advise on interpretation or writing up of results. There are no plans to disseminate the results of the research to study participants or the relevant patient community.

## Results

### Observational analyses: higher levels of education are associated with myopia

**Fig 2:**
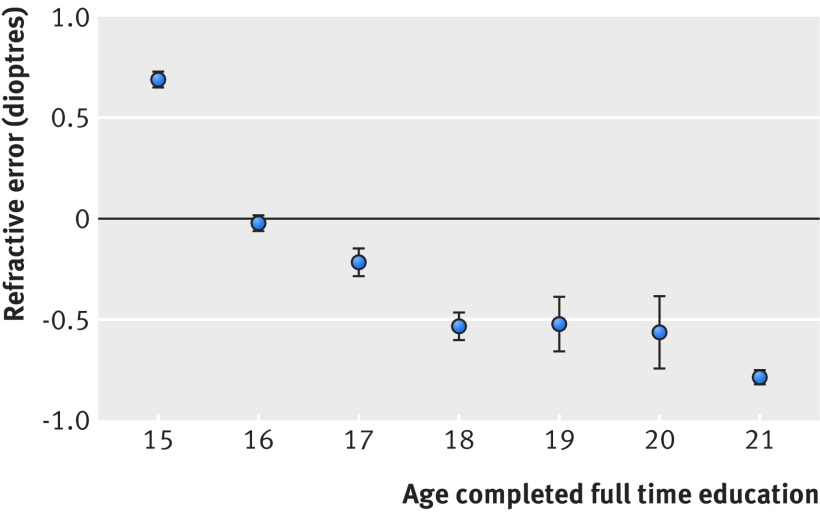
Observational association between age full time education was completed and refractive error for 69 798 people in UK Biobank. On average, more educated people had higher levels of myopia (more negative refractive error). Whiskers represent 95% confidence intervals

In agreement with previous studies,^6^ ^13^ ^30^ participants in UK Biobank who had spent longer in full time education were more myopic; that is, they had increasingly negative refractive errors (table 1). The relation was linear for those leaving full time education between the ages of 15 and 18 years, meaning that every additional year in education was associated with a higher myopic refractive error by −0.25 dioptres/y. For those leaving full time education after age 18 years, the rate slowed to −0.10 dioptres/y (fig 2). On average, every additional year spent in education was associated with a more myopic refractive error of −0.18 dioptres/y (95% confidence interval −0.19 to −0.17, P<2e-16). The association was largely unaffected by adjustment for measured potential confounders, including socioeconomic position (Townsend deprivation index), birth weight, breast fed, and place of birth (northing and easting coordinates; table 1).

**Table 1:** Observational association between time spent in education and refractive error

Exposure	Outcome	Model A*				Model B†		
		No of participants	Effect size	P value		No of participants	Effect size	P value
Time spent in education‡	Refractive error§	69 798	−0.178 (−0.185 to −0.170) (dioptres/y)	<2e-16		37 734	−0.165 (−0.179 to −0.154) (dioptres/y)	<2e-16
Refractive error§	Time spent in education‡	69 798	−0.154 (−0.161 to −0.147) (y/dioptre)	<2e-16		37 734	−0.136 (−0.145 to −0.128) (y/dioptre)	<2e-16

* Included sex and age as covariates.

† Included age, sex, Townsend deprivation index, birth weight, whether breast fed, and northing and easting coordinates.

‡ Age full time education was completed (in years).

§ Average measured mean spherical equivalent refractive error (in dioptres).

### Mendelian randomisation analyses: more time spent in education causes myopia

Bidirectional mendelian randomisation was used to assess the causality and direction of the association between time spent in education and refractive error. Bidirectional analyses consist of two separate mendelian randomisation calculations—one in each direction. Firstly, to calculate the causal effect of education on myopia we used a weighted education allele score as the instrumental variable. Secondly, to calculate the causal effect of myopia on time spent in education we used a weighted myopia allele score as the instrumental variable. We derived the allele score for time spent in education from genetic variants identified by Okbay et al^34^ in a large meta-analysis of GWAS of people of European descent (n=293 723). Likewise, we derived the allele score for myopia from genetic variants reported by Pickrell et al^15^ in a GWAS of self reported myopia (n=191 843).

The myopia allele score explained 4.32% (*F*=3155) of the variance in average mean spherical equivalent refractive error of participants in UK Biobank and the education allele score explained 0.71% (*F*=464) of the variance in time spent in education. We selected these genetic variants to use as instrumental variables because of their robust association with time spent in education and myopia, allowing us to construct strong aggregate instrumental variables for making mendelian randomisation inferences. The large F statistics suggested that these analyses would not be affected by weak instrument bias.

**Fig 3:**
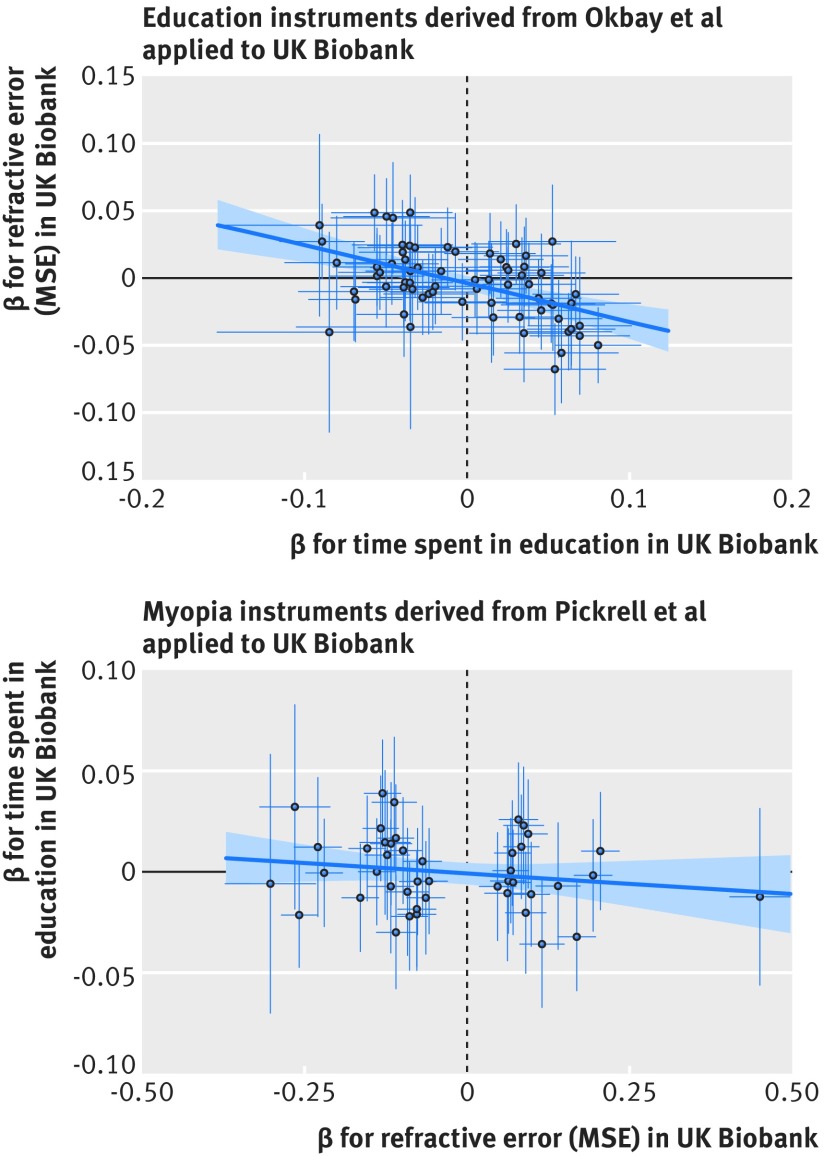
Results of bidirectional mendelian randomisation. (Top panel) 69 variants associated with educational attainment in Okbay et al^34^ were linked to higher levels of myopia (more negative mean spherical equivalent (MSE)) in UK Biobank. (Bottom panel) 44 variants associated with myopia (more negative MSE) in Pickrell et al^15^ were not linked with more time spent in education in UK Biobank. Regression line and standard errors (shaded area) fitted using robust linear regression. Whiskers represent 95% confidence intervals

Thus, using the allele score for time spent in education as the instrumental variable, mendelian randomisation analysis showed that every additional year spent in education resulted in a more myopic refractive error of −0.27 dioptres/y (95% confidence interval −0.37 to −0.17, P=4e-8) (table 2; fig 3). The mendelian randomisation effect estimate was even greater in magnitude than the observational estimate (−0.27 *v* −0.18 dioptres) suggesting that unmeasured confounders may have attenuated the latter relation. Conversely, using the myopia allele score as the instrumental variable in mendelian randomisation analyses provided little evidence that refractive error affected time spent in education (β_IV_=−0.008 y/dioptre, 95% confidence interval −0.041 to 0.025, P=0.6) (table 2; fig 3). With a sample size of n=69 798, there was 80% power to detect an effect of time spent in education on refractive error ≥0.14 dioptres/y. In the reciprocal direction, there was 80% power to detect an effect ≥0.048 y/dioptre (see supplementary figure 1), suggesting that this study had sufficient power to detect an effect of myopia on education, if present.

**Table 2:** Causal association between time spent in education and refractive error. Results of conventional multivariable linear regression and bidirectional mendelian randomisation. All regressions included age and sex as covariates

Exposure	Outcome	No of participants	Observational estimate (OLS): effect size	Mendelian randomisation regression			
				Partial R^2^	P value (DWH)	Effect size	P value
Time spent in education*	Refractive error†	69 798	−0.178 (−0.185 to −0.170) (dioptres/y)	0.71%	0.06	−0.270 (−0.368 to −0.173) (dioptres/y)	4e-8
Refractive error†	Time spent in education*	69 798	−0.154 (−0.161 to −0.147) (y/dioptre)	4.32%	<2e-16	−0.008 (−0.041 to 0.025) (y/dioptre)	0.6

OLS=ordinary least squares; DWH=Durbin-Wu-Hausman test for endogeneity.

* Age full time education was completed (in years).

† Average measured mean spherical equivalent refractive error (in dioptres).

### Sensitivity analyses: results of mendelian randomisation are robust to potential bias

#### Confounding

Mendelian randomisation analyses are based on two pertinent assumptions: the genetic instrumental variables are not associated with any confounders of the exposure-outcome relation, and the genetic instrumental variables are only associated with the outcome through the exposure.

**Fig 4:**
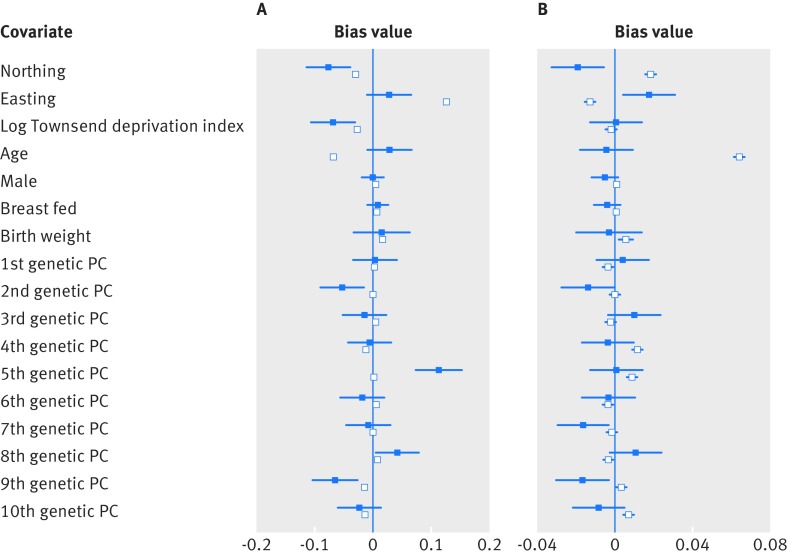
Confounding bias plots. Plots showing relative bias in instrumental variable estimate (blue) and standard multivariable regression estimate (white) from potential confounders including: place of birth (northing and easting coordinates), Townsend deprivation index, age, sex, breastfed, birth weight, and first 10 genetic principal components (PC), when **(**A) estimating the effect of time spent in education on refractive error; and (B) estimating the effect of refractive error on time spent in education. Townsend deprivation index was natural log transformed

In tests of the association between the allele scores for time spent in education and myopia with potential confounders, there was evidence that the geographical coordinate, northing (measured northward distance in UK) was negatively associated with time spent in education (β=−1.6e-6, 95% confidence interval −1.8e-6 to −1.5e-6) and positively with refractive error (β=1.2e-6, 9.8e-7 to 1.3e-6). Northing was also associated with the time spent in education (P=7e-5) and myopia (P=6e-3) allele scores (see supplementary table 2). Compared with standard regression, the confounding bias plot suggested that inclusion of the northing variable in the instrumental variable analysis would result in a greater degree of bias for the education allele score but not for the myopia allele score (fig 4).

In contrast, the geographical easting coordinate was positively associated with time spent in education (β=8.9e-7, 95% confidence interval 6.8e-7 to 1.1e-6) and negatively associated with refractive error (β=−1.0e-6, −1.3e-6 to −8.1e-6). It was weakly associated with the myopia allele score (P=0.01). However, there was little evidence to suggest a greater degree of bias in the instrumental variable analysis compared with a standard regression with the inclusion of the easting variable (fig 4). Sensitivity analyses suggested that confounding bias from the geographical coordinates had negligible impact on the mendelian randomisation results (see supplementary table 4).

One further confounding variable, population stratification principal component 9 (PC9), incurred a greater degree of bias in the instrumental variable regression compared with observational least squares regression. Additional analyses showed that PC9 was associated with a self reported place of birth in Wales (see supplementary figure S2) and also with a −0.17 dioptre (95% confidence interval −0.05 to −0.28) more myopic refractive error, on average (P=4e-3) compared with those who reported being born in England. A mendelian randomisation sensitivity analysis that adjusted for population stratification principal components 1-10 provided similar results to those before adjustment (see supplementary table 4), suggesting that confounding due to PC9 did not lead to appreciable bias.

While education legislation has not been different in England and Wales while the UK Biobank participants were in education, Scottish schools normally finish one year earlier and university degrees are correspondingly one year longer. This difference would impact on the years spent in education for Scottish people moving to England to attend university, and vice versa. However, the results of a mendelian randomisation sensitivity analysis restricted to participants born in England were essentially unchanged (see supplementary table 4), providing evidence that imprecision in quantifying years spent in education due to differences in school leaving age did not adversely affect the results.

#### Horizontal (genetic) pleiotropy

Under the second assumption of mendelian randomisation, genetic variants with pleiotropic effects are invalid instrumental variables. This can be problematic when genetic variants are used without regard for the biological mechanisms through which they affect the exposure—for example, if the genetic variants associated with more years in education also caused myopia independently of the education phenotype. Mendelian randomisation-Egger, weighted mode, and weighted median methods are alternative methods of integrating instrumental variable estimates across individual single nucleotide polymorphisms. These methods allow some of the assumptions of mendelian randomisation to be relaxed providing valid tests for causality despite the presence of invalid instrumental variables (eg, due to genetic pleiotropy).^54^ If the results across different mendelian randomisation methods are divergent, this may indicate that genetic pleiotropy is creating bias. However, all methods yielded similar causal estimates in magnitude and direction, such that increasing time spent in education led to a more myopic refractive error (by −0.17 to −0.40 dioptres/y), whereas there was little evidence that a more myopic refractive error led to more time spent in education (see supplementary table 3). With mendelian randomisation-Egger, a deviation of the intercept estimate from zero suggests the existence of genetic pleiotropy—that is, where certain genetic variants affect the outcome through a different biological pathway from the exposure under investigation. In practice, there was little evidence that the Egger intercept deviated from zero either for more time in education causing refractive error (intercept=0.007, SE=0.006, P=0.2) or refractive error causing more time in education (intercept=−0.002, SE=0.007, P=0.8), indicating that there was little evidence for directional genetic pleiotropy.

#### Measurement error

Encoding time spent in education as a dichotomous trait (>16 versus ≤16 years of age when completed full time education) produced the same pattern of causality as the continuous variable, age at which full time education was completed—that is, more time spent in education had an effect on refractive error (β_IV_=−0.35 dioptres/LOD(education) where LOD is the logarithm of odds for having spent >16 versus ≤16 years in education, 95% confidence interval −0.48 to −0.22), whereas refractive error did not have an effect on time spent in education (β_IV_=−0.0004 LOD(education)/dioptre, −0.03 to 0.03) (see supplementary table 4).

When those who had attended university or college were excluded from the analyses, there was a similar point estimate of the effect of time spent in education on refractive error (β_IV_=−0.23 dioptres/y, 95% confidence interval −0.48 to 0.02, P=0.07) with larger standard errors. This was attributable, in part, to the reduced sample size (n=45 535). Again, there was little evidence that refractive error had an effect on time spent in education (β_IV_=−0.004 y/dioptre, 95% confidence interval −0.04 to 0.03, P=0.8) (see supplementary table 4).

Using the Durbin-Wu-Hausman test for endogeneity, we found weak evidence that the instrumental variable estimate using the time spent in education allele score differed from the observational point estimate (Durbin-Wu-Hausman P=0.06), with the instrumental variable estimate suggesting a larger negative association (table 2). There was strong evidence that the instrumental variable estimate using the myopia allele score was a departure from the observational point estimate (Durbin-Wu-Hausman P<2e-16; table 2).

## Discussion

In this study, we found strong evidence that more time spent in education is a causal risk factor for myopia. More specifically, every additional year in education caused an increase in myopic refractive error of −0.27 dioptres/y (95% confidence interval −0.37 to −0.17, P=4e-8). Thus the cumulative effect of more years in education on refractive error means that someone attending university would be likely to have at least −1 dioptre more myopia than someone who left school at age 16. A difference of this magnitude would blur vision on a Snellen visual acuity chart to 6/18 and affect the ability to drive without glasses. Those with myopia, by definition, have better near vision than distance vision and require less accommodative effort for near work and study, and so myopia has been proposed as an educational advantage.^55^ Despite the general perception that people with myopia are more studious than those without myopia, there was little evidence that being myopic resulted in people remaining in education for longer.

### Strengths and limitations of this study

Mendelian randomisation is a particularly powerful approach for testing causal hypotheses in epidemiology.^56^ ^57^ The large sample size and robustly associated genetic instrumental variables used here meant that causal effects could be estimated with high precision. Consistent with other studies, the allele score for myopia explained only a small fraction (4.32%) of the variance in refractive error of participants in UK Biobank.^20^ Likewise, the education allele score explained only a small fraction of the variance (0.71%) in time spent in education of participants in UK Biobank.^34^ However, power calculations confirmed these effects were sufficient to draw solid inferences from the results of mendelian randomisation analysis presented here (see supplementary figure S1). Given the ubiquity of exposure to education in populations with available genotype data, it is not possible to assess those who were completely free of the outcome, specifically education. Nor is it ethical to randomise children to different levels of or years in education to assess the impact on refractive error. The advantage of mendelian randomisation is that participants are grouped based on their genotype—randomly allocated at conception and so analogous to a randomised controlled trial in which genetic variants are used as proxies for an environmental exposure to make causal inferences about the impact of the exposure on the outcome of interest. However, it is not possible to determine exactly which components of educational practices in the past 5-7 decades have led to increases in myopic refraction using mendelian randomisation. Although more robust to confounding than standard observational studies, mendelian randomisation is not entirely immune. There was some evidence of confounding by the variables northing and PC9*,* of which the latter identified people from Wales. Although there are some differences between the education system in England and Wales compared with Scotland, the results held true when analyses were restricted to people from England. Another limitation of this study was selection bias. UK Biobank participants have been shown to be more highly educated, have healthier lifestyles, and have fewer self reported adverse health outcomes than expected compared with the general UK population.^58^ This selection bias could have led to bias in both the observational and the mendelian randomisation effect estimates.^59^

When using mendelian randomisation it is not necessary to know how the genetic variants used in the analysis cause the exposure. Yet, without knowing the function of the genetic variants and how they influence the traits described here, it is possible that some single nucleotide polymorphisms may influence the outcome through a pathway that does not involve the exposure—that is, through horizontal pleiotropy. For example, educational outcomes and intelligence are highly correlated, and if intelligence caused myopia through a pathway that did not involve exposure to education, this could cause bias in the mendelian randomisation causal effect estimate (see supplementary figure 3a). In contrast, vertical pleiotropy refers to single nucleotide polymorphisms that influence the outcome through an intermediate phenotype—for example, if some single nucleotide polymorphisms affect exposure to education through their influence on intelligence. Vertical pleiotropy acting through intelligence would not bias the mendelian randomisation causal estimate obtained here (see supplementary figure 3b). Sensitivity analyses (mendelian randomisation-Egger and mode based mendelian randomisation; see supplementary table S3) suggested little evidence of unbalanced horizontal pleiotropy in the relation between education and myopia, although such bias cannot be ruled out unequivocally.

### Comparison with other studies

In agreement with a substantial number of epidemiological studies dating back more than 100 years,^6^ ^13^ ^30^ the observational analyses in this study showed that more highly educated participants in UK Biobank were more myopic. The results of bidirectional mendelian randomisation analyses showed that this association arises from exposure to factors related to education on myopia. The current epidemic of myopia in developed East and South-East Asian countries over the past one or two generations seems to coincide with widening exposure to primary and secondary education, whereas educational outcomes (eg, in scientific, reading, and mathematical literacy) are less clearly associated with myopia, since many Western countries achieve top international rankings in student assessments without the same high prevalence rates of myopia.^60^ Moreover, there are countries with poorly developed education systems in which the prevalence of myopia is low,^61^ ^62^ ^63^ ^64^ and hence any causal relation between intelligence and myopia is unlikely. There are other well established associations between myopia and urbanisation, reduced light exposure, socioeconomic position, near work, and prenatal factors,^21^ ^65^ ^66^ ^67^ and several of these factors either confound the relation between education and myopia or may work synergistically to exacerbate the effect—for example, in countries where myopia prevalence is particularly high. Despite the robust associations between exposure to education and myopia reported by many of these previous studies, they have not shown causality. Only one study has addressed the causal relation between education and myopia: in a mendelian randomisation analysis of three cohorts of European ancestry (combined n=5649), Cuellar-Partida et al^68^ reported that each year of education led to a more myopic refractive error of −0.46 dioptres/y (P=1e-3). However, the study was under-powered and the authors did not investigate the possibility of horizontal genetic pleiotropy or reverse causation.^68^ Moreover, their methodology risked violating the key assumptions of mendelian randomisation because they used several thousand single nucleotide polymorphisms (n=17 749) to construct a polygenic risk score as an instrumental variable for their measure of education. The number of single nucleotide polymorphisms used in this previous study means it was more likely to include pleiotropic variants with direct effects both on exposure to education and on refractive error, and single nucleotide polymorphisms that are in linkage disequilibrium with refractive error variants. The much larger sample size in this study permitted the use of a small number of strongly associated variants as instrumental variables for exposure to education and refractive error. Thus the risk of linkage disequilibrium between the major risk variants for the two traits explaining the underlying associations between education and myopia was mitigated. Crucially, the analyses in this study provided strong evidence that the relation arose from a causal effect of exposure to education on refractive error, and not through reverse causation or confounding by influences such as socioeconomic position.

Exactly how increasing levels of education cause myopia cannot be inferred from mendelian randomisation analyses, although the known environmental risk factors for myopia provide intriguing clues. Children from developed East and South-East Asian countries consistently report that they spend less time outdoors than children from Australia or the United States,^25^ ^27^ ^28^ ^69^ ^70^ ^71^ ^72^ ^73^ and randomised controlled trials have found that time spent outdoors during childhood protects against the development of myopia.^24^ ^25^ Therefore, lack of time outdoors is a plausible mediator in the causal pathway linking more time spent in education and myopia. Furthermore, engaging in higher levels of near work activities, such as reading, is associated with the incidence and progression of myopia, albeit less consistently than time spent outdoors.^11^ ^26^ ^73^ ^74^ ^75^ Yet, measures of time spent on near work activities and time spent outdoors are generally uncorrelated.^27^ ^28^ ^29^ Thus, lack of time outdoors and excessive near work may not be the only routes mediating the effects of exposure to education on myopia. Children with myopia tend to engage in less physical activity, such as sports, but physical activity in itself does not seem to be protective.^29^ ^76^ Others have correlated higher light exposure with lower myopia risk,^66^ ^77^ and it is possible that those who spend more years in education have less exposure to natural light. The progression of myopia is faster in winter months, thus supporting the hypothesis that exposure to natural light is important.^78^ ^79^ This hypothesis has been one of the main drivers for recent investment in “bright light classrooms” to protect against myopia in South-East Asia.^80^ Whether these classrooms provide any protection against myopia that replicates the effects of increasing time spent outdoors is not currently known as the impact of this intervention has not yet been measured. The best recommendation, based on the highest quality available evidence at the moment, is for children to spend more time outside (www.nhs.uk/conditions/short-sightedness/).

### Conclusions and policy implications

This study provides strong evidence that more time spent in education is a causal risk factor for myopia. With the rapid increase in the global prevalence of myopia and the economic burden of myopia and its vision threatening complications, the findings of this study have important implications for educational practices. Axial eye growth occurs predominantly during the school years^81^ and since levels of myopia tend to stabilise in adulthood^23^ any interventions to halt or prevent myopia need to be delivered in childhood. Policy makers should be aware that the educational practices used to educate children and to promote personal and economic health may have the unintended consequence of causing increasing levels of myopia and later visual disability.

**What is already known on this topic**

Myopia, or short-sightedness, is a leading cause of visual disability worldwide, and the global prevalence is increasing rapidly
Numerous observational studies have reported strong associations between educational outcomes and myopia
Because randomising children to different levels of education would be unethical it was not known whether increasing exposure to education causes myopia, myopic children are more studious, or socioeconomic position leads to myopia and higher levels of education

**What this study adds**

More time spent in education is a causal risk factor for myopia
This study highlights a need for further research and discussion about how educational practices might be improved to achieve better outcomes without adversely affecting vision

## Web extra

Extra material supplied by authors

## Notes

Ethical approval: The UK Biobank Resource and Access Committee approved this research (application #8786). Anonymised phenotype and genetic data are available from UK Biobank on application.

Data sharing: Participants consented to data sharing as described in the population cohorts and are not identifiable in these analyses. Code implementing the statistical methods to analyse the data are available from https://github.com/edm1/myopia-education-MR.

Transparency: The corresponding author (DA) is guarantor of the paper and affirms that the manuscript is an honest, accurate and transparent account of the study being reported; that no important aspects of the study have been omitted; and that any discrepancies from the study as planned have been explained.
