# Supplementary material for: Education and myopia: assessing the direction of causality by mendelian randomisation
Source: BMJ. 2018 Jun 6;361:k2022. doi: 10.1136/bmj.k2022 (PMC5987847; doi:10.1136/bmj.k2022)
Supplement: Supplementary file 1 — Appendix: Supplementary figures and tables [file moue042661.ww1.pdf]

Supplementary Figures and Tables

Supplementary Figure 1. Mendelian randomisation power calculation for the available sample size in UK Biobank (N=69,798). Causal association of: **(A)** time spent in education (years [yrs]) on refractive error (Dioptres [D]) using allele score with R^2=0.0073 (80% power = 0.14 D/yr); and **(B)** refractive error on time spent in education using allele score with R^2=0.0442 (80% power = 0.048 yr/D).

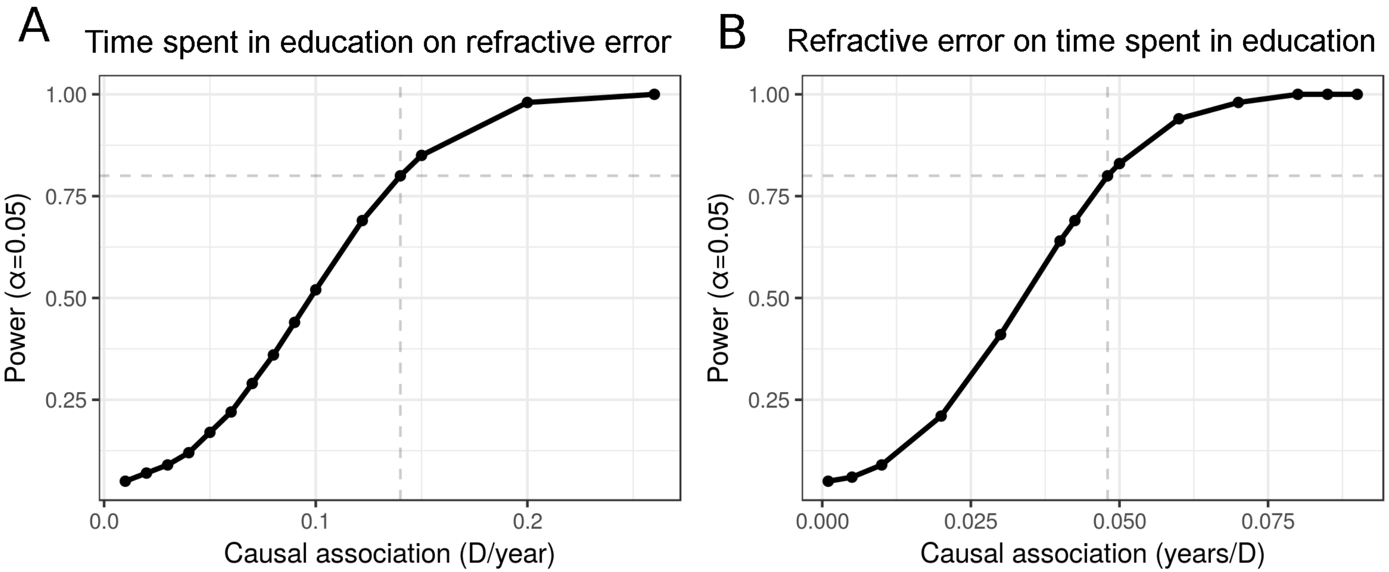

Supplementary Figure 2. Genetic principal component 9 (PC9) is associated with self-reported place-of-birth in Wales and with refractive error. (A) Scatter plot of geographic coordinates for place-of-birth vs. PC9 level. **(B)** Refractive error distribution in Dioptres (D) by PC9 level category. White rectangle shows inter-quartile range. Black circle shows median.

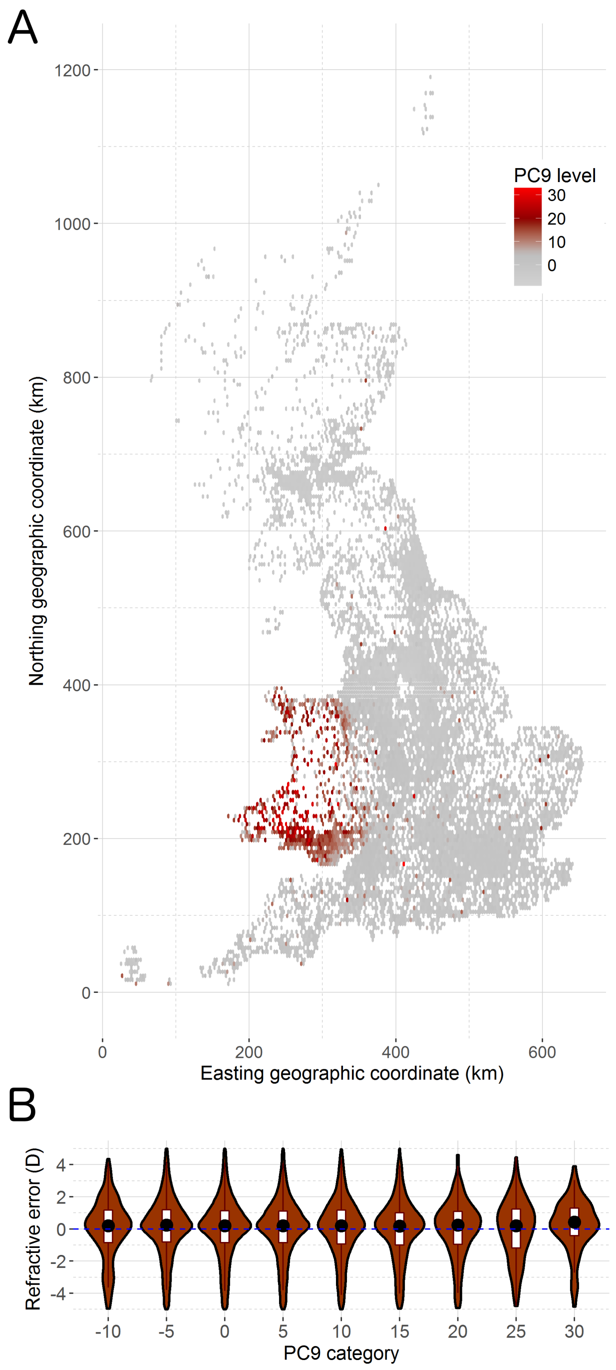

Supplementary Figure 3. Schematic representations of horizontal and vertical pleiotropy in the relationship between the instrumental variables, education and IQ. (A) Horizontal pleiotropy could lead to bias in the MR causal estimate, but only if there is a causal path between IQ and myopia. In communities with no formal education system there is a very low prevalence of myopia, making such a causal path between IQ and myopia unlikely. **(B)** Vertical pleiotropy would not lead to bias in the MR causal estimate.

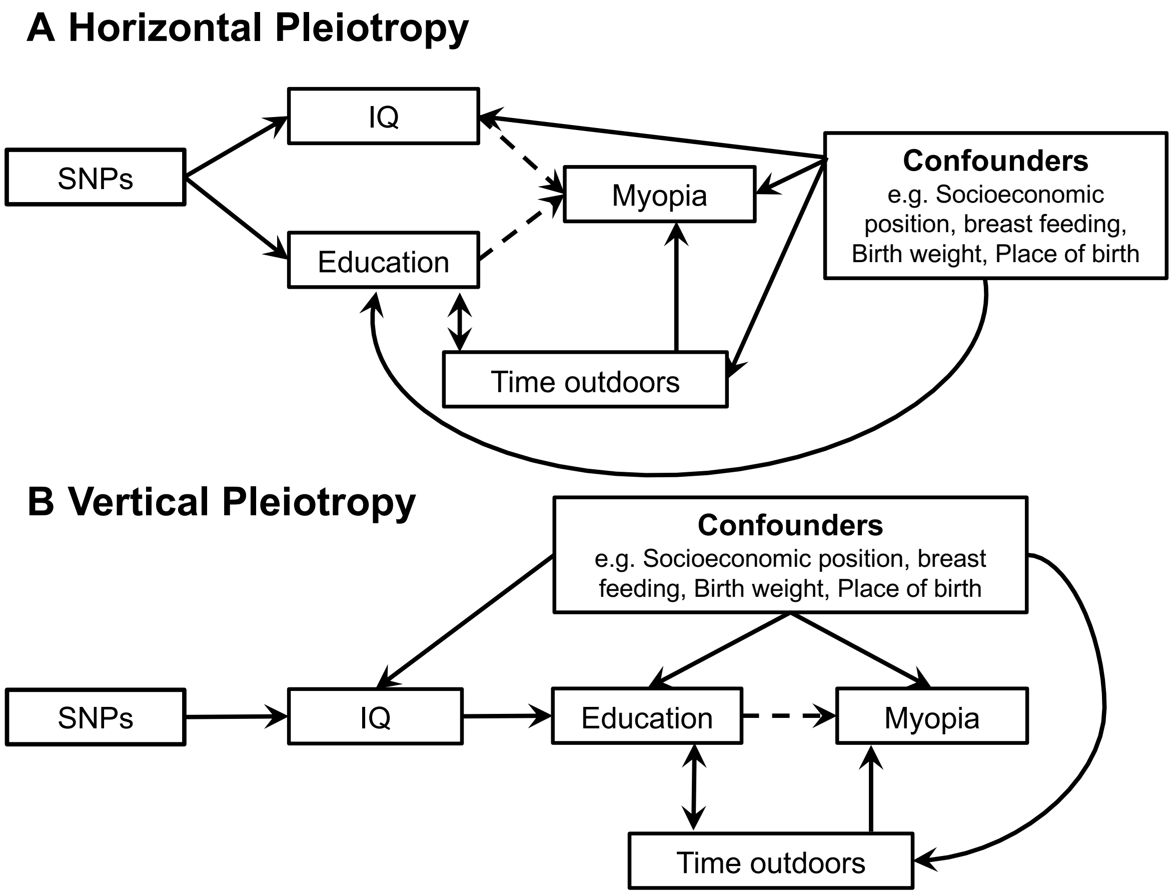

Supplementary Table 1. Baseline characteristics of UK Biobank cohort for refractive error, age completed education and potential confounder variables.

Refractive errors (in Dioptres) were defined as: (i) myopia ≤ 0.75; (ii) -0.75 < emmetropia < 0.75; (iii) hypermetropia ≥ 0.75.

Townsend deprivation index (TDI) was natural log-transformed.

**Variable**	**All** (N=69,798)		**Myopic** (N=21,055)		**Emmetropic** (N=25,209)		**Hypermetropic** (N=23,534)	
	**Mean or %**	**SD**	**Mean or %**	**SD**	**Mean or %**	**SD**	**Mean or %**	**SD**
Refractive error (D)	-0.271 (-0.291 to -0.251)	2.679	-3.329 (-3.361 to -3.297)	2.385	0.071 (0.066 to 0.076)	0.400	2.099 (2.081 to 2.117)	1.385
Age completed education (years)	18.15 (18.13 to 18.17)	2.50	18.80 (18.76 to 18.83)	2.39	18.08 (18.05 to 18.11)	2.46	17.66 (17.62 to 17.69)	2.51
Age (years)	57.09 (57.03 to 57.14)	7.81	55.97 (55.86 to 56.07)	7.70	55.14 (55.04 to 55.24)	8.10	60.18 (60.09 to 60.26)	6.56
Sex								
- Male	47.2%	-	47.4%	-	48.6%	-	45.5%	-
- Female	52.8%	-	52.6%	-	51.4%	-	54.5%	-
Birth weight	3.325 (3.320 to 3.330)	0.637	3.310 (3.302 to 3.318)	0.622	3.330 (3.322 to 3.338)	0.628	3.332 (3.324 to 3.340)	0.662
Breastfed								
- Yes	78.6%	-	78.3%	-	87.9%	-	81.6%	-
- No	20.4%	-	20.7%	-	22.1%	-	18.4%	-
Townsend deprivation index	-0.012 (-0.016 to -0.008)	0.495	-0.012 (-0.019 to -0.005)	0.493	-0.005 (-0.011 to 0.001)	0.494	-0.019 (-0.025 to -0.013)	0.499
Northing	3.016e+5 (3.007e+5 to 3.024e+5)	1.135e+5	2.955e+5 (2.030e+5 to 2.971e+5)	1.162e+5	3.015e+5 (3.001e+5 to 3.029e+5)	1.124e+5	3.071e+5 (3.057e+5 to 3.085e+5)	1.121e+5
Easting	4.279e+5 (4.272e+5 to 4.286e+5)	8.911e+4	4.318e+5 (4.305e+5 to 4.330e+5)	9.089e+4	4.318e+5 (4.305e+5 to 4.330e+5)	9.089e+4	4.224e+5 (4.233e+5 to 4.256e+5)	8.869e+4

Supplementary Table 2. Observational (OLS) associations of confounding variables with education and myopia allele scores and outcomes.

Only associations that are concordant with the observational direction of effect between time spent in education and refractive error are shown. These include *northing* and *easting* geographical coordinates, and genetic population stratification principal components 8 and 9.

Exposure	Outcome	N	Beta	SE	P-value
Easting	Refractive error	69797	-1.0e-6 (-1.3e-6 to -8.1e-7)	1.1e-7	<2e-16
Easting	Education	69797	8.9e-7 (6.8e-7 to 1.1e-6)	1.1e-7	<2e-16
Easting	Myopia allele score	69797	3.8e-8 (8.6e-9 to 6.7e-8)	1.5e-8	1e-2
Easting	Education allele score	69797	6.1e-9 (-2.2e-9 to 1.4e-8)	4.2e-9	2e-1
Northing	Education	69797	-1.6e-6 (-1.8e-6 to -1.5e-6)	8.3e-8	<2e-16
Northing	Refractive error	69797	1.2e-6 (9.8e-7 to 1.3e-6)	8.9e-8	<2e-16
Northing	Education allele score	69797	-1.3e-8 (-2.0e-8 to -6.7e-9)	3.3e-9	7e-5
Northing	Myopia allele score	69797	-3.2e-8 (-5.5e-8 to -9.2e-9)	1.2e-8	6e-3
PC8	Education	69798	2.7e-2 (1.7e-2 to 3.7e-2)	4.9e-3	3e-8
PC8	Refractive error	69798	-1.2e-2 (-2.3e-2 to -2.2e-3)	5.2e-3	2e-2
PC8	Education allele score	69798	4.2e-4 (4.2e-5 to 8.1e-4)	1.9e-4	3e-2
PC8	Myopia allele score	69798	1.1e-3 (-2.9e-4 to 2.4e-3)	6.9e-4	1e-1
PC9	Education	69798	-2.1e-2 (-2.5e-2 to -1.7e-2)	2.3e-3	<2e-16
PC9	Education allele score	69798	-3.1e-4 (-4.8e-4 to -1.3e-4)	9.1e-5	8e-4
PC9	Myopia allele score	69798	-7.8e-4 (-1.4e-3 to -1.5e-4)	3.2e-4	2e-2
PC9	Refractive error	69798	5.6e-3 (8.0e-4 to 1.0e-2)	2.4e-3	2e-2

Supplementary Table 3. Causal estimates of time spent in education on refractive error and refractive error on time spent in education using methods implemented in MR-Base and a split sample in UK Biobank.

Exposure	*Outcome*	*Method*	*N SNPs*	*Beta*	*SE*	*P-value*
Time spent in education	Refractive error	MR Egger	67	-0.399	0.125	2e-3
		Weighted median	67	-0.206	0.099	0.04
		Inverse variance weighted	67	-0.271	0.070	1e-4
		Simple mode	67	-0.169	0.547	0.8
		Weighted mode	67	-0.231	0.529	0.7
Refractive error	Time spent in education	MR Egger	43	-0.002	0.057	1
		Weighted median	43	0.003	0.037	0.9
		Inverse variance weighted	43	-0.014	0.026	0.6
		Simple mode	43	-0.091	0.071	0.2
		Weighted mode	43	0.030	0.049	0.6

Supplementary Table 4. Table of MR results from sensitivity analyses using alternative encodings of educational exposure and outcome, additional covariates and England-only restriction.

Instrument	Exposure	Outcome	Covariates	N	TSLS Beta	TSLS SE	TSLS P	Weak instr P	DWH P
Education	Education	Refractive error	sex + age	69798	-0.270 (-0.368 to -0.173)	0.049	4e-8	<2e-16	6e-2
Education	Education (England only)	Refractive error	sex + age	68152	-0.269 (-0.369 to -0.171)	0.050	8e-8	<2e-16	7e-2
Education	Education	Refractive error	sex + age + genechip	69798	-0.270 (-0.368 to -0.173)	0.049	4e-8	<2e-16	6e-2
Education	Education	Refractive error	sex + age + PC1-10	69798	-0.277 (-0.376 to -0.179)	0.050	3e-8	<2e-16	4e-2
Education	Education	Refractive error	sex + age + birthweight	42511	-0.272 (-0.399 to -0.146)	0.064	2e-5	<2e-16	1e-1
Education	Education	Refractive error	sex + age + breastfed	53601	-0.307 (-0.417 to -0.200)	0.055	2e-8	<2e-16	1e-2
Education	Education	Refractive error	sex + age + northing	69797	-0.264 (-0.363 to -0.166)	0.050	1e-7	<2e-16	7e-2
Education	Education	Refractive error	sex + age + easting	69797	-0.268 (-0.366 to -0.171)	0.049	5e-8	<2e-16	6e-2
Education	Education	Refractive error	sex + age + TDI	69737	-0.271 (-0.371 to -0.173)	0.050	6e-8	<2e-16	6e-2
Education	Education (Dichotomised)	Refractive error	sex + age	69798	-0.347 (-0.482 to -0.220)	0.064	-	-	-
Education	Education (No college)	Refractive error	sex + age	45535	-0.228 (-0.479 to 0.018)	0.124	7e-2	<2e-16	5e-1
Myopia	Refractive error	Education	sex + age	69798	-0.008 (-0.041 to 0.025)	0.017	6e-1	<2e-16	<2e-16
Myopia	Refractive error	Education (England only)	sex + age	68152	-0.009 (-0.042 to 0.025)	0.017	6e-1	<2e-16	<2e-16
Myopia	Refractive error	Education (No college)	sex + age	45535	-0.004 (-0.035 to 0.027)	0.016	8e-1	<2e-16	7e-06
Myopia	Refractive error	Education (Dichotomised)	sex + age	69798	0.000 (-0.028 to 0.028)	0.014	-	-	-
Myopia	Refractive error	Education	sex + age + genechip	69798	-0.009 (-0.041 to 0.025)	0.017	6e-1	<2e-16	<2e-16
Myopia	Refractive error	Education	sex + age + PC1-10	69798	-0.011 (-0.044 to 0.022)	0.017	5e-1	<2e-16	<2e-16
Myopia	Refractive error	Education	sex + age + birthweight	42511	0.016 (-0.026 to 0.057)	0.021	5e-1	<2e-16	6e-15
Myopia	Refractive error	Education	sex + age + breastfed	53601	0.007 (-0.030 to 0.044)	0.019	7e-1	<2e-16	<2e-16
Myopia	Refractive error	Education	sex + age + northing	69797	-0.011 (-0.044 to 0.022)	0.017	5e-1	<2e-16	<2e-16
Myopia	Refractive error	Education	sex + age + easting	69797	-0.009 (-0.042 to 0.024)	0.017	6e-1	<2e-16	<2e-16
Myopia	Refractive error	Education	sex + age + TDI	69737	-0.008 (-0.040 to 0.026)	0.017	7e-1	<2e-16	<2e-16

Supplementary Data Table. Genetic variants used as instrumental variables for time spent in education and myopia in this study. Summary statistics are shown for associations with (1) time spent in education/myopia in the original study, (2) time spent in education and refractive error in the full UK Biobank sample, (3) time spent in education and refractive error in the two UK Biobank split sample groups.

Myopia SNPs (Pickrell *et al.*, 2016)

					Association with myopia in Pickrell *et al.* (2016)						Association with time spent in education in full UKBB cohort							Association with refractive error in full UKBB cohort				Association with time spent in education in UKBB split sample group A				Association with refractive error in UKBB split sample group A				Association with time spent in education in UKBB split sample group B				Association with refractive error in UKBB split sample group B			
SNPID	Chrom	Pos	Genes	Cyto	Pickrell Effect allele	Pickrell Other allele	logOR	logOR_lowerCI	logOR_upperCI	P	UKBB Effect allele	UKBB Other allele	EAF	Beta	SE	P	N	Beta	Se	P	N	Beta	SE	P	N	Beta	SE	P	N	Beta	SE	P	N	Beta	SE	P	N
1:61341632_T_A	1	61341632	C1orf87[]NFIA	1p31.3	A	T	-0.056	-0.070	0.008	5.70E-13	A	T	0.327	0.005	0.014	7.18E-01	69798	-0.069	0.015	5.48E-06	69798	0.004	0.020	8.6E-01	34982	-0.091	0.021	1.9E-05	34982	0.007	0.020	7.4E-01	34816	-0.047	0.022	3.0E-02	34816
1:164213686_C_T	1	164213686	NUF2[]PBX1	1q23.3	C	T	0.077	0.060	0.009	2.70E-19	T	C	0.242	0.014	0.015	3.70E-01	69798	-0.118	0.017	1.39E-12	69798	0.048	0.022	2.7E-02	34982	-0.117	0.023	4.5E-07	34982	-0.022	0.022	3.2E-01	34816	-0.118	0.024	5.8E-07	34816
1:200336075_C_T	1	200336075	NR5A2[]ZNF281	1q32.1	C	T	0.063	0.048	0.008	3.10E-17	T	C	0.377	-0.022	0.014	1.03E-01	69798	-0.088	0.015	1.23E-09	69798	-0.009	0.019	6.5E-01	34982	-0.093	0.020	4.6E-06	34982	-0.036	0.019	6.3E-02	34816	-0.083	0.021	5.8E-05	34816
2:45152748_T_C	2	45152748	CAMKMT[]SIX3	2p21	C	T	0.063	0.045	0.009	7.80E-12	C	T	0.188	0.011	0.017	5.26E-01	69798	0.062	0.018	6.08E-04	69798	-0.004	0.024	8.6E-01	34982	0.031	0.026	2.3E-01	34982	-0.017	0.024	4.8E-01	34816	0.094	0.026	2.7E-04	34816
2:146888708_G_A	2	146888708	[]	2q22.3	A	G	0.068	0.053	0.008	3.40E-20	A	G	0.442	-0.011	0.013	3.96E-01	69798	0.099	0.014	4.28E-12	69798	-0.017	0.019	3.7E-01	34982	0.106	0.020	1.0E-07	34982	-0.006	0.019	7.7E-01	34816	0.092	0.020	7.5E-06	34816
2:157358750_T_A	2	157358750	[GPD2]	2q24.1	A	T	0.059	0.043	0.008	5.80E-13	A	T	0.697	0.026	0.014	7.34E-02	69798	0.079	0.015	3.00E-07	69798	0.042	0.020	4.1E-02	34982	0.072	0.022	9.1E-04	34982	0.009	0.020	6.5E-01	34816	0.086	0.022	9.3E-05	34816
2:172851936_C_G	2	172851936	HAT1[]METAP1D	2q31.1	C	G	-0.067	-0.084	0.009	3.20E-15	G	C	0.236	-0.020	0.015	1.85E-01	69798	0.090	0.016	4.12E-08	69798	-0.022	0.022	3.0E-01	34982	0.075	0.023	1.2E-03	34982	-0.018	0.022	4.1E-01	34816	0.105	0.023	7.0E-06	34816
rs17400325	2	178565913	[PDE11A]	2q31.2	C	T	-0.170	-0.206	0.018	9.60E-21	C	T	0.040	-0.006	0.033	8.55E-01	69798	-0.302	0.035	1.22E-17	69798	-0.020	0.047	6.6E-01	34982	-0.235	0.050	2.5E-06	34982	0.008	0.046	8.7E-01	34816	-0.366	0.050	2.2E-13	34816
rs1550094	2	233385396	[PRSS56]	2q37.1	A	G	0.097	0.082	0.008	9.20E-35	A	G	0.692	-0.002	0.014	9.01E-01	69798	0.193	0.015	1.43E-36	69798	-0.008	0.020	6.9E-01	34982	0.188	0.021	1.7E-18	34982	0.004	0.020	8.4E-01	34816	0.199	0.022	1.0E-19	34816
3:24268677_C_T	3	24268677	[THRB]	3p24.2	C	T	-0.052	-0.066	0.007	2.30E-12	T	C	0.619	-0.007	0.014	5.92E-01	69798	0.047	0.015	1.39E-03	69798	-0.028	0.019	1.5E-01	34982	0.043	0.021	3.8E-02	34982	0.013	0.019	4.9E-01	34816	0.051	0.021	1.5E-02	34816
3:141076084_T_C	3	141076084	[ZBTB38]	3q23	C	T	0.067	0.052	0.008	7.70E-19	C	T	0.389	-0.005	0.013	7.01E-01	69798	0.071	0.014	9.57E-07	69798	-0.013	0.019	5.0E-01	34982	0.055	0.020	6.6E-03	34982	0.002	0.019	9.2E-01	34816	0.087	0.021	2.5E-05	34816
4:80508788_T_C	4	80508788	GK2[]ANTXR2	4q21.21	C	T	0.066	0.050	0.008	2.60E-15	C	T	0.729	0.023	0.015	1.29E-01	69798	0.087	0.016	5.79E-08	69798	0.010	0.021	6.3E-01	34982	0.105	0.023	3.4E-06	34982	0.035	0.021	9.8E-02	34816	0.070	0.023	2.3E-03	34816
6:28270584_T_C	6	28270584	PGBD1[]ZSCAN31	6p22.1	C	T	-0.059	-0.075	0.008	1.20E-12	C	T	0.299	-0.018	0.015	2.05E-01	69798	-0.078	0.016	6.17E-07	69798	-0.009	0.020	6.5E-01	34982	-0.061	0.022	5.4E-03	34982	-0.027	0.021	1.9E-01	34816	-0.096	0.022	1.9E-05	34816
6:50809720_T_C	6	50809720	[TFAP2B]	6p12.3	C	T	0.050	0.035	0.007	1.50E-11	C	T	0.456	-0.004	0.013	7.33E-01	69798	0.064	0.014	7.12E-06	69798	0.001	0.019	9.6E-01	34982	0.062	0.020	1.9E-03	34982	-0.010	0.019	6.0E-01	34816	0.064	0.020	1.5E-03	34816
6:73643289_C_A	6	73643289	[KCNQ5]	6q13	A	C	-0.124	-0.138	0.007	1.90E-63	A	C	0.590	0.000	0.013	9.77E-01	69798	-0.219	0.014	6.54E-52	69798	-0.013	0.019	4.9E-01	34982	-0.204	0.020	7.3E-24	34982	0.012	0.019	5.2E-01	34816	-0.235	0.021	5.1E-30	34816
rs1064583	6	116446576	[NT5DC1:COL10A1]	6q22.1	A	G	0.054	0.040	0.007	1.60E-13	G	A	0.402	-0.005	0.013	7.18E-01	69798	-0.076	0.014	1.48E-07	69798	-0.004	0.019	8.5E-01	34982	-0.083	0.020	4.3E-05	34982	-0.005	0.019	7.8E-01	34816	-0.070	0.021	7.1E-04	34816
rs12193446	6	129820038	[LAMA2]	6q22.33	A	G	-0.263	-0.286	0.012	5.40E-102	G	A	0.092	-0.012	0.022	5.78E-01	69798	0.450	0.024	1.41E-78	69798	0.021	0.032	5.1E-01	34982	0.450	0.034	6.3E-41	34982	-0.045	0.032	1.5E-01	34816	0.450	0.034	2.2E-39	34816
8:40723970_A_G	8	40723970	[ZMAT4]	8p11.21	A	G	-0.102	-0.120	0.009	4.00E-31	G	A	0.205	-0.007	0.016	6.64E-01	69798	0.139	0.017	1.50E-15	69798	-0.021	0.023	3.6E-01	34982	0.112	0.024	4.1E-06	34982	0.007	0.023	7.6E-01	34816	0.167	0.025	2.6E-11	34816
rs10089517	8	60178721	TOX[]CA8	8q12.1	A	C	0.102	0.087	0.007	6.00E-41	A	C	0.343	-0.032	0.014	2.05E-02	69798	0.168	0.015	7.12E-30	69798	-0.030	0.019	1.2E-01	34982	0.152	0.021	2.4E-13	34982	-0.034	0.020	8.0E-02	34816	0.184	0.021	3.1E-18	34816
9:18362865_A_G	9	18362865	SH3GL2[]ADAMTSL1	9p22.2	A	G	0.056	0.042	0.007	2.30E-14	G	A	0.586	0.017	0.014	2.21E-01	69798	-0.110	0.015	4.43E-14	69798	0.016	0.019	4.1E-01	34982	-0.108	0.020	1.2E-07	34982	0.017	0.019	3.9E-01	34816	-0.111	0.021	8.5E-08	34816
9:71770939_G_A	9	71770939	FXN[]TJP2	9q21.11	A	G	-0.083	-0.101	0.009	6.70E-21	A	G	0.212	-0.010	0.016	5.41E-01	69798	-0.091	0.017	1.02E-07	69798	-0.009	0.023	6.9E-01	34982	-0.104	0.024	1.7E-05	34982	-0.011	0.023	6.3E-01	34816	-0.080	0.024	1.1E-03	34816
10:60339098_A_C	10	60339098	[BICC1]	10q21.1	A	C	-0.056	-0.069	0.007	2.80E-14	C	A	0.541	0.012	0.013	3.49E-01	69798	0.083	0.014	5.04E-09	69798	0.023	0.019	2.3E-01	34982	0.075	0.020	1.6E-04	34982	0.002	0.019	9.0E-01	34816	0.091	0.020	8.1E-06	34816
10:79063542_T_A	10	79063542	[KCNMA1]	10q22.3	A	T	-0.074	-0.088	0.007	3.30E-22	A	T	0.358	0.000	0.014	9.90E-01	69798	-0.139	0.015	2.06E-21	69798	-0.023	0.019	2.2E-01	34982	-0.135	0.020	4.1E-11	34982	0.024	0.019	2.1E-01	34816	-0.143	0.021	8.0E-12	34816
10:114788436_C_T	10	114788436	[TCF7L2]	10q25.2	C	T	0.075	0.057	0.009	1.10E-16	T	C	0.198	0.008	0.016	6.12E-01	69798	-0.123	0.018	2.89E-12	69798	0.020	0.023	3.8E-01	34982	-0.140	0.025	1.4E-08	34982	-0.004	0.023	8.7E-01	34816	-0.106	0.025	2.4E-05	34816
rs1550870	11	18751041	[PTPN5]	11p15.1	C	T	-0.051	-0.066	0.008	9.90E-13	T	C	0.460	0.009	0.013	4.79E-01	69798	0.069	0.014	9.34E-07	69798	-0.005	0.019	8.0E-01	34982	0.057	0.020	4.1E-03	34982	0.024	0.019	2.0E-01	34816	0.082	0.020	4.6E-05	34816
11:40149300_G_A	11	40149300	[LRRC4C]	11p12	A	G	-0.168	-0.188	0.010	9.20E-66	A	G	0.169	0.012	0.018	4.84E-01	69798	-0.230	0.019	7.12E-34	69798	0.030	0.025	2.3E-01	34982	-0.238	0.027	2.8E-19	34982	-0.006	0.025	8.2E-01	34816	-0.221	0.027	3.3E-16	34816
11:84634790_C_A	11	84634790	[DLG2]	11q14.1	A	C	-0.076	-0.090	0.007	2.30E-25	A	C	0.479	0.011	0.013	4.30E-01	69798	-0.098	0.014	7.12E-12	69798	-0.008	0.019	6.8E-01	34982	-0.090	0.020	7.6E-06	34982	0.029	0.019	1.2E-01	34816	-0.106	0.020	1.9E-07	34816
11:117672561_C_T	11	117672561	DSCAML1[]FXYD6FXYD2	11q23.3	C	T	0.050	0.035	0.007	1.40E-11	T	C	0.413	-0.005	0.013	7.32E-01	69798	-0.058	0.014	4.70E-05	69798	0.003	0.019	8.6E-01	34982	-0.067	0.020	8.2E-04	34982	-0.013	0.019	5.0E-01	34816	-0.049	0.020	1.6E-02	34816
11:131931531_G_A	11	131931531	[NTM]	11q25	A	G	0.059	0.045	0.007	9.70E-16	A	G	0.423	0.001	0.013	9.66E-01	69798	0.067	0.014	2.55E-06	69798	-0.010	0.019	5.8E-01	34982	0.070	0.020	4.7E-04	34982	0.011	0.019	5.5E-01	34816	0.064	0.020	1.6E-03	34816
rs5442	12	6954864	[GNB3]	12p13.31	A	G	-0.111	-0.139	0.014	2.30E-14	A	G	0.067	0.032	0.026	2.14E-01	69798	-0.265	0.028	1.30E-21	69798	-0.003	0.036	9.3E-01	34982	-0.300	0.039	1.1E-14	34982	0.067	0.037	6.6E-02	34816	-0.230	0.040	6.7E-09	34816
12:9313304_A_G	12	9313304	[PZP]	12p13.31	A	G	0.055	0.039	0.008	3.80E-12	G	A	0.308	-0.021	0.014	1.35E-01	69798	-0.078	0.015	3.41E-07	69798	-0.057	0.020	4.1E-03	34982	-0.054	0.021	1.1E-02	34982	0.015	0.020	4.7E-01	34816	-0.101	0.022	3.1E-06	34816
rs3138141	12	56115778	[RDH5]	12q13.2	A	C	0.136	0.119	0.009	1.50E-51	A	C	0.244	0.010	0.015	4.95E-01	69798	0.205	0.016	2.29E-37	69798	0.021	0.021	3.2E-01	34982	0.213	0.022	1.7E-21	34982	-0.001	0.021	9.7E-01	34816	0.195	0.023	2.1E-17	34816
13:100689354_G_A	13	100689354	ZIC2[]PCCA	13q32.3	A	G	-0.075	-0.089	0.007	8.90E-25	A	G	0.440	0.021	0.013	1.06E-01	69798	-0.133	0.014	1.17E-20	69798	0.011	0.019	5.5E-01	34982	-0.146	0.020	3.5E-13	34982	0.032	0.019	9.1E-02	34816	-0.120	0.020	2.8E-09	34816
14:42273570_C_G	14	42273570	[LRFN5]	14q21.1	C	G	0.078	0.059	0.010	2.00E-16	G	C	0.188	0.034	0.017	3.83E-02	69798	-0.112	0.018	3.25E-10	69798	0.013	0.024	5.7E-01	34982	-0.085	0.025	6.8E-04	34982	0.055	0.023	1.8E-02	34816	-0.138	0.025	5.3E-08	34816
14:54552428_A_T	14	54552428	BMP4[]CDKN3	14q22.2	A	T	0.076	0.058	0.009	4.70E-17	T	A	0.294	-0.030	0.014	3.60E-02	69798	-0.109	0.015	1.30E-12	69798	-0.034	0.020	9.1E-02	34982	-0.114	0.022	1.2E-07	34982	-0.026	0.020	2.1E-01	34816	-0.105	0.022	1.8E-06	34816
15:35005886_T_A	15	35005886	GOLGA8B[]GJD2	15q14	A	T	-0.121	-0.135	0.007	3.50E-62	A	T	0.494	-0.021	0.013	1.04E-01	69798	-0.258	0.014	1.67E-74	69798	-0.025	0.019	1.8E-01	34982	-0.269	0.020	1.2E-41	34982	-0.017	0.019	3.5E-01	34816	-0.247	0.020	1.1E-34	34816
15:63571234_C_T	15	63571234	[APH1B]	15q22.2	C	T	-0.059	-0.076	0.009	1.70E-11	T	C	0.221	-0.036	0.016	2.68E-02	69798	0.115	0.017	2.74E-11	69798	-0.015	0.023	5.0E-01	34982	0.105	0.024	1.7E-05	34982	-0.056	0.023	1.4E-02	34816	0.125	0.025	3.4E-07	34816
15:79378821_C_T	15	79378821	[RASGRF1]	15q25.1	C	T	0.064	0.050	0.007	4.30E-18	T	C	0.416	0.012	0.013	3.85E-01	69798	-0.153	0.014	6.66E-27	69798	0.009	0.019	6.4E-01	34982	-0.150	0.020	5.7E-14	34982	0.014	0.019	4.6E-01	34816	-0.157	0.020	1.4E-14	34816
16:7459347_T_A	16	7459347	[RBFOX1]	16p13.3	A	T	-0.127	-0.142	0.008	2.00E-63	A	T	0.359	-0.013	0.014	3.50E-01	69798	-0.164	0.015	1.83E-28	69798	0.013	0.019	5.0E-01	34982	-0.180	0.021	5.2E-18	34982	-0.039	0.020	4.4E-02	34816	-0.147	0.021	3.1E-12	34816
17:11407259_T_A	17	11407259	[SHISA6]	17p12	A	T	-0.076	-0.090	0.007	5.30E-24	A	T	0.397	0.039	0.014	4.27E-03	69798	-0.130	0.015	5.22E-19	69798	0.049	0.019	9.9E-03	34982	-0.146	0.020	9.6E-13	34982	0.028	0.019	1.4E-01	34816	-0.113	0.021	4.7E-08	34816
17:31239645_C_T	17	31239645	MYO1D[]TMEM98	17q11.2	C	T	0.063	0.045	0.009	3.80E-12	T	C	0.191	-0.007	0.017	6.70E-01	69798	-0.117	0.018	1.22E-10	69798	-0.011	0.024	6.3E-01	34982	-0.110	0.026	1.7E-05	34982	-0.002	0.024	9.2E-01	34816	-0.124	0.026	1.5E-06	34816
17:79606820_T_C	17	79606820	NPLOC4[]TSPAN10	17q25.3	C	T	0.071	0.055	0.008	1.60E-18	C	T	0.654	0.019	0.014	1.71E-01	69798	0.094	0.015	1.50E-10	69798	-0.001	0.019	9.4E-01	34982	0.081	0.021	8.4E-05	34982	0.039	0.019	4.2E-02	34816	0.108	0.021	2.8E-07	34816
rs12965607	18	47391025	[MYO5B]	18q21.1	G	T	-0.086	-0.106	0.011	2.40E-16	G	T	0.148	0.015	0.018	4.25E-01	69798	-0.126	0.020	1.41E-10	69798	0.021	0.026	4.2E-01	34982	-0.131	0.027	1.8E-06	34982	0.009	0.026	7.4E-01	34816	-0.120	0.028	1.9E-05	34816
21:47371947_G_A	21	47371947	PCBP3[]COL6A1	21q22.3	A	G	-0.058	-0.074	0.008	5.10E-13	A	G	0.301	-0.013	0.014	3.67E-01	69798	-0.063	0.015	3.76E-05	69798	-0.021	0.020	3.0E-01	34982	-0.068	0.022	1.6E-03	34982	-0.005	0.020	8.2E-01	34816	-0.059	0.022	7.7E-03	34816

Education SNPs (Okbay *et al.*, 2016)

				Association with time spent in education in Okbay *et al.* (2016)								Association with time spent in education in full UKBB cohort					Association with refractive error in full UKBB cohort			
RSID	SNPID	Chrom	Pos	Okbay Effect allele	Okbay Other allele	Okbay EAF	Beta	SE	P	UKBB Effect allele	UKBB Other allele	UKBB EAF	Beta	SE	P	N	Beta	SE	P	N
rs301800	rs301800	1	8490603	T	C	0.1791	0.019	0.003	1.79E-08	C	T	0.824	-0.070	0.017	5.86E-05	69798	-0.010	0.019	5.88E-01	69798
rs11210860	1:43982527_G_A	1	43982527	A	G	0.3694	0.017	0.003	2.36E-10	A	G	0.371	0.056	0.014	3.25E-05	69798	-0.031	0.015	3.66E-02	69798
rs34305371	rs34305371	1	72733610	A	G	0.08769	0.035	0.005	3.76E-14	A	G	0.098	0.064	0.022	3.16E-03	69798	-0.018	0.023	4.29E-01	69798
rs2568955	1:72762169_T_C	1	72762169	T	C	0.2369	-0.017	0.003	1.80E-08	C	T	0.741	0.054	0.016	6.66E-04	69798	-0.068	0.017	7.23E-05	69798
rs1008078	1:91189731_C_T	1	91189731	T	C	0.3731	-0.016	0.003	6.01E-10	T	C	0.390	-0.057	0.013	2.29E-05	69798	0.048	0.014	8.16E-04	69798
rs11588857	1:204587047_G_A	1	204587047	A	G	0.209	0.02	0.003	5.27E-10	A	G	0.208	0.053	0.016	1.05E-03	69798	-0.020	0.017	2.51E-01	69798
rs1777827	1:211613114_G_A	1	211613114	A	G	0.569	0.015	0.003	1.55E-08	A	G	0.594	0.013	0.013	3.17E-01	69798	-0.001	0.014	9.45E-01	69798
rs2992632	1:243503764_A_T	1	243503764	A	T	0.6604	0.017	0.003	8.23E-09	T	A	0.289	-0.046	0.015	1.60E-03	69798	0.011	0.016	4.93E-01	69798
rs76076331	2:10977585_C_T	2	10977585	T	C	0.09328	0.02	0.004	3.63E-08	T	C	0.121	0.053	0.020	8.67E-03	69798	0.027	0.022	2.05E-01	69798
rs11689269	2:15621917_C_G	2	15621917	C	G	0.306	0.016	0.003	1.28E-08	G	C	0.668	-0.024	0.014	9.40E-02	69798	-0.012	0.015	4.38E-01	69798
rs1606974	2:51873599_A_G	2	51873599	A	G	0.1101	0.022	0.004	2.80E-08	G	A	0.880	-0.046	0.020	2.08E-02	69798	0.044	0.021	3.58E-02	69798
rs11690172	2:57387094_A_G	2	57387094	A	G	0.6119	0.015	0.003	1.99E-08	G	A	0.402	-0.021	0.013	1.16E-01	69798	-0.011	0.014	4.66E-01	69798
rs2457660	2:60757419_C_T	2	60757419	T	C	0.6063	-0.017	0.003	7.11E-10	T	C	0.633	-0.040	0.014	3.81E-03	69798	0.019	0.015	1.96E-01	69798
rs10496091	rs10496091	2	61482261	A	G	0.2705	-0.018	0.003	5.62E-10	A	G	0.277	-0.039	0.015	8.31E-03	69798	-0.027	0.016	8.54E-02	69798
rs13402908	2:100333377_T_C	2	100333377	T	C	0.5056	-0.018	0.003	1.70E-11	C	T	0.525	0.036	0.013	6.28E-03	69798	0.016	0.014	2.48E-01	69798
rs4851251	2:100753490_C_T	2	100753490	T	C	0.2537	-0.017	0.003	1.91E-08	T	C	0.268	-0.031	0.015	3.94E-02	69798	0.008	0.016	6.33E-01	69798
rs12987662	2:100821548_C_A	2	100821548	A	C	0.3787	0.027	0.003	2.69E-24	A	C	0.393	0.067	0.013	6.47E-07	69798	-0.012	0.014	4.02E-01	69798
rs17824247	2:144152539_T_C	2	144152539	T	C	0.5802	-0.016	0.003	2.77E-09	C	T	0.403	0.025	0.013	6.09E-02	69798	-0.005	0.014	7.31E-01	69798
rs16845580	2:161920884_T_C	2	161920884	T	C	0.6306	0.016	0.003	2.65E-09	C	T	0.377	-0.049	0.014	2.98E-04	69798	0.046	0.015	1.78E-03	69798
rs4500960	2:162818621_C_T	2	162818621	T	C	0.4776	-0.016	0.003	3.75E-10	T	C	0.481	-0.035	0.013	7.09E-03	69798	0.024	0.014	9.10E-02	69798
rs6739979	2:193731929_T_C	2	193731929	T	C	0.597	-0.015	0.003	4.70E-08	C	T	0.366	0.046	0.014	9.28E-04	69798	0.004	0.015	8.11E-01	69798
rs2245901	2:194296294_G_A	2	194296294	A	G	0.4216	-0.016	0.003	4.54E-09	A	G	0.401	-0.034	0.013	1.20E-02	69798	-0.008	0.014	5.57E-01	69798
rs55830725	2:237056854_T_A	2	237056854	A	T	0.1679	-0.022	0.004	5.37E-10	A	T	0.167	-0.032	0.018	6.94E-02	69798	0.022	0.019	2.38E-01	69798
rs35761247	rs35761247	3	48623124	A	G	0.05597	0.034	0.006	3.82E-08	A	G	0.057	0.069	0.028	1.29E-02	69798	-0.043	0.030	1.51E-01	69798
rs62259535	3:48939052_A_G	3	48939052	A	G	0.97015	0.048	0.008	2.63E-09	G	A	0.033	-0.085	0.035	1.61E-02	69798	-0.040	0.038	2.88E-01	69798
rs11712056	3:49914397_T_C	3	49914397	T	C	0.5504	0.024	0.003	3.30E-19	C	T	0.436	-0.089	0.013	1.50E-11	69798	0.027	0.014	5.71E-02	69798
rs112634398	3:50075494_A_G	3	50075494	A	G	0.95896	0.036	0.007	4.61E-08	G	A	0.045	-0.091	0.032	4.83E-03	69798	0.039	0.035	2.60E-01	69798
rs62263923	3:85674790_A_G	3	85674790	A	G	0.6437	-0.016	0.003	7.00E-09	G	A	0.351	0.052	0.014	1.84E-04	69798	-0.019	0.015	2.08E-01	69798
rs6799130	3:160847801_C_G	3	160847801	C	G	0.5131	-0.015	0.003	2.82E-08	G	C	0.482	0.016	0.013	2.12E-01	69798	-0.029	0.014	3.80E-02	69798
rs12646808	4:3249828_T_C	4	3249828	T	C	0.6418	0.016	0.003	4.00E-08	C	T	0.343	-0.012	0.014	3.93E-01	69798	0.023	0.015	1.34E-01	69798
rs2610986	4:18037231_C_T	4	18037231	T	C	0.6511	-0.016	0.003	2.01E-08	T	C	0.653	0.014	0.014	3.26E-01	69798	0.018	0.015	2.37E-01	69798
rs34072092	4:28801221_T_C	4	28801221	T	C	0.8694	0.024	0.004	3.91E-08	C	T	0.108	0.015	0.021	4.72E-01	69798	-0.019	0.023	4.07E-01	69798
rs3101246	rs3101246	4	42649935	T	G	0.5821	-0.015	0.003	1.43E-08	T	G	0.604	-0.003	0.013	8.30E-01	69798	-0.018	0.014	2.19E-01	69798
rs4863692	4:140764124_G_T	4	140764124	T	G	0.334	0.018	0.003	1.56E-10	T	G	0.330	0.030	0.014	3.20E-02	69798	0.025	0.015	9.39E-02	69798
rs4493682	5:45188024_G_C	5	45188024	C	G	0.2034	0.019	0.004	3.32E-08	C	G	0.188	0.035	0.017	4.09E-02	69798	-0.041	0.019	2.65E-02	69798
rs2964197	5:57535206_C_T	5	57535206	T	C	0.5019	0.015	0.003	3.02E-08	T	C	0.487	0.032	0.013	1.41E-02	69798	-0.029	0.014	4.21E-02	69798
rs61160187	5:60111579_A_G	5	60111579	A	G	0.6194	-0.017	0.003	3.49E-10	G	A	0.382	0.062	0.013	3.60E-06	69798	-0.040	0.014	6.08E-03	69798
rs324886	5:87896602_C_T	5	87896602	T	C	0.3601	-0.015	0.003	1.91E-08	T	C	0.374	-0.038	0.014	5.03E-03	69798	0.014	0.015	3.48E-01	69798
rs10061788	5:87934707_G_A	5	87934707	A	G	0.2164	0.021	0.004	2.46E-09	A	G	0.177	0.058	0.018	1.10E-03	69798	-0.056	0.019	3.60E-03	69798
rs2431108	5:103947968_T_C	5	103947968	T	C	0.6325	0.016	0.003	5.27E-09	C	T	0.323	-0.035	0.014	1.24E-02	69798	0.005	0.015	7.31E-01	69798
rs1402025	5:113987898_T_C	5	113987898	T	C	0.7481	0.017	0.003	3.42E-08	C	T	0.222	-0.040	0.016	1.17E-02	69798	0.025	0.017	1.45E-01	69798
rs62379838	5:120102028_T_C	5	120102028	T	C	0.681	0.016	0.003	3.30E-08	C	T	0.297	-0.050	0.014	4.79E-04	69798	-0.006	0.015	6.81E-01	69798
rs56231335	6:98187291_T_C	6	98187291	T	C	0.6474	-0.017	0.003	2.07E-09	C	T	0.321	0.035	0.014	1.28E-02	69798	0.009	0.015	5.72E-01	69798
rs7767938	6:153367613_T_C	6	153367613	T	C	0.7705	0.017	0.003	2.44E-08	C	T	0.247	-0.016	0.015	2.97E-01	69798	0.005	0.016	7.65E-01	69798
rs2615691	7:23402104_G_A	7	23402104	A	G	0.02799	-0.037	0.007	4.71E-08	A	G	0.039	-0.035	0.036	3.30E-01	69798	-0.036	0.038	3.44E-01	69798

	Association with time spent in education in UKBB split sample group A				Association with refractive error in UKBB split sample group A				Association with time spent in education in UKBB split sample group B				Association with refractive error in UKBB split sample group B			
RSID	Beta	SE	P	N	Beta	SE	P	N	Beta	SE	P	N	Beta	SE	P	N
rs301800	-0.109	0.024	7.2E-06	34982	-0.009	0.026	7.3E-01	34982	-0.029	0.025	2.3E-01	34816	-0.011	0.027	6.9E-01	34816
rs11210860	0.043	0.019	2.4E-02	34982	-0.039	0.020	5.8E-02	34982	0.069	0.019	3.3E-04	34816	-0.022	0.021	3.0E-01	34816
rs34305371	0.079	0.031	1.0E-02	34982	-0.013	0.033	6.9E-01	34982	0.049	0.031	1.1E-01	34816	-0.024	0.033	4.8E-01	34816
rs2568955	0.026	0.022	2.5E-01	34982	-0.074	0.024	2.0E-03	34982	0.082	0.022	2.6E-04	34816	-0.060	0.024	1.3E-02	34816
rs1008078	-0.064	0.019	7.6E-04	34982	0.053	0.020	9.0E-03	34982	-0.050	0.019	8.4E-03	34816	0.044	0.021	3.3E-02	34816
rs11588857	0.057	0.023	1.2E-02	34982	-0.003	0.024	9.0E-01	34982	0.048	0.023	3.5E-02	34816	-0.038	0.025	1.2E-01	34816
rs1777827	0.030	0.019	1.1E-01	34982	0.002	0.020	9.2E-01	34982	-0.003	0.019	8.6E-01	34816	-0.004	0.021	8.3E-01	34816
rs2992632	-0.044	0.021	3.6E-02	34982	-0.008	0.022	7.2E-01	34982	-0.049	0.021	1.7E-02	34816	0.030	0.022	1.8E-01	34816
rs76076331	0.019	0.028	5.0E-01	34982	0.058	0.030	5.2E-02	34982	0.086	0.028	2.6E-03	34816	-0.005	0.031	8.8E-01	34816
rs11689269	-0.037	0.020	6.1E-02	34982	-0.003	0.021	8.9E-01	34982	-0.010	0.020	6.3E-01	34816	-0.020	0.022	3.4E-01	34816
rs1606974	-0.020	0.028	4.8E-01	34982	0.065	0.030	2.9E-02	34982	-0.072	0.028	9.6E-03	34816	0.023	0.030	4.5E-01	34816
rs11690172	-0.039	0.019	3.9E-02	34982	-0.019	0.020	3.4E-01	34982	-0.003	0.019	8.6E-01	34816	-0.001	0.021	9.5E-01	34816
rs2457660	-0.034	0.019	8.3E-02	34982	0.017	0.021	4.2E-01	34982	-0.047	0.019	1.6E-02	34816	0.022	0.021	3.0E-01	34816
rs10496091	-0.050	0.021	1.6E-02	34982	-0.047	0.022	3.4E-02	34982	-0.027	0.021	1.9E-01	34816	-0.007	0.023	7.5E-01	34816
rs13402908	0.035	0.019	6.1E-02	34982	0.029	0.020	1.5E-01	34982	0.037	0.019	5.1E-02	34816	0.003	0.020	8.7E-01	34816
rs4851251	-0.011	0.021	5.8E-01	34982	0.009	0.022	6.8E-01	34982	-0.050	0.021	1.8E-02	34816	0.007	0.023	7.7E-01	34816
rs12987662	0.065	0.019	6.8E-04	34982	0.004	0.020	8.4E-01	34982	0.069	0.019	3.0E-04	34816	-0.030	0.021	1.5E-01	34816
rs17824247	0.028	0.019	1.4E-01	34982	0.001	0.020	9.7E-01	34982	0.022	0.019	2.4E-01	34816	-0.012	0.020	5.7E-01	34816
rs16845580	-0.052	0.019	6.9E-03	34982	0.068	0.020	8.7E-04	34982	-0.047	0.019	1.4E-02	34816	0.024	0.021	2.6E-01	34816
rs4500960	-0.042	0.019	2.5E-02	34982	0.043	0.020	3.1E-02	34982	-0.029	0.019	1.2E-01	34816	0.006	0.020	7.8E-01	34816
rs6739979	0.070	0.020	3.7E-04	34982	-0.022	0.021	2.9E-01	34982	0.022	0.019	2.6E-01	34816	0.029	0.021	1.7E-01	34816
rs2245901	-0.052	0.019	5.5E-03	34982	-0.016	0.020	4.3E-01	34982	-0.015	0.019	4.4E-01	34816	-0.002	0.021	9.2E-01	34816
rs55830725	-0.026	0.025	3.0E-01	34982	0.023	0.027	3.9E-01	34982	-0.038	0.025	1.4E-01	34816	0.021	0.027	4.4E-01	34816
rs35761247	0.078	0.039	4.5E-02	34982	-0.024	0.042	5.7E-01	34982	0.059	0.040	1.4E-01	34816	-0.065	0.043	1.3E-01	34816
rs62259535	-0.101	0.050	4.3E-02	34982	-0.018	0.053	7.3E-01	34982	-0.070	0.050	1.6E-01	34816	-0.063	0.054	2.4E-01	34816
rs11712056	-0.083	0.019	7.4E-06	34982	0.018	0.020	3.6E-01	34982	-0.095	0.019	3.6E-07	34816	0.037	0.020	7.1E-02	34816
rs112634398	-0.042	0.045	3.5E-01	34982	0.058	0.048	2.3E-01	34982	-0.139	0.046	2.2E-03	34816	0.020	0.049	6.9E-01	34816
rs62263923	0.040	0.020	4.0E-02	34982	-0.036	0.021	8.2E-02	34982	0.064	0.020	1.2E-03	34816	-0.001	0.021	9.7E-01	34816
rs6799130	0.012	0.019	5.2E-01	34982	-0.045	0.020	2.4E-02	34982	0.020	0.019	2.7E-01	34816	-0.014	0.020	4.8E-01	34816
rs12646808	-0.013	0.020	5.0E-01	34982	0.016	0.021	4.6E-01	34982	-0.010	0.020	6.0E-01	34816	0.029	0.022	1.8E-01	34816
rs2610986	-0.002	0.020	9.0E-01	34982	0.014	0.021	5.1E-01	34982	0.031	0.020	1.3E-01	34816	0.023	0.022	2.8E-01	34816
rs34072092	0.033	0.030	2.7E-01	34982	-0.028	0.032	3.8E-01	34982	-0.002	0.030	9.5E-01	34816	-0.010	0.032	7.5E-01	34816
rs3101246	0.003	0.019	8.6E-01	34982	-0.021	0.020	2.9E-01	34982	-0.010	0.019	6.2E-01	34816	-0.013	0.021	5.2E-01	34816
rs4863692	0.019	0.020	3.3E-01	34982	0.028	0.021	1.8E-01	34982	0.040	0.020	4.2E-02	34816	0.023	0.021	2.9E-01	34816
rs4493682	0.049	0.024	4.5E-02	34982	-0.022	0.026	4.0E-01	34982	0.021	0.024	3.9E-01	34816	-0.060	0.026	2.2E-02	34816
rs2964197	0.032	0.019	9.0E-02	34982	-0.029	0.020	1.4E-01	34982	0.034	0.019	7.1E-02	34816	-0.028	0.020	1.6E-01	34816
rs61160187	0.062	0.019	1.1E-03	34982	-0.052	0.020	1.1E-02	34982	0.063	0.019	9.0E-04	34816	-0.028	0.021	1.7E-01	34816
rs324886	-0.017	0.019	3.9E-01	34982	-0.012	0.020	5.4E-01	34982	-0.060	0.019	1.9E-03	34816	0.040	0.021	5.7E-02	34816
rs10061788	0.070	0.025	5.4E-03	34982	-0.066	0.027	1.3E-02	34982	0.046	0.025	6.9E-02	34816	-0.045	0.027	1.0E-01	34816
rs2431108	-0.032	0.020	1.1E-01	34982	-0.007	0.021	7.4E-01	34982	-0.038	0.020	5.2E-02	34816	0.018	0.021	4.1E-01	34816
rs1402025	-0.035	0.022	1.2E-01	34982	0.024	0.024	3.1E-01	34982	-0.045	0.022	4.2E-02	34816	0.025	0.024	3.0E-01	34816
rs62379838	-0.075	0.020	1.9E-04	34982	0.050	0.021	2.1E-02	34982	-0.025	0.020	2.2E-01	34816	-0.062	0.022	4.5E-03	34816
rs56231335	0.064	0.020	1.3E-03	34982	0.006	0.021	7.9E-01	34982	0.006	0.020	7.8E-01	34816	0.012	0.022	5.7E-01	34816
rs7767938	-0.032	0.022	1.4E-01	34982	0.006	0.023	7.9E-01	34982	0.000	0.022	9.9E-01	34816	0.004	0.024	8.6E-01	34816
rs2615691	-0.088	0.051	8.2E-02	34982	-0.017	0.054	7.5E-01	34982	0.021	0.051	6.8E-01	34816	-0.054	0.055	3.2E-01	34816

rs12531458	7:39090698_A_C	7	39090698	A	C	0.5354	0.014	0.003	3.11E-08	C	A	0.481	-0.035	0.013	8.18E-03	69798	0.049	0.014	6.07E-04	69798	-0.074	0.019	8.1E-05	34982	0.054	0.020	6.2E-03	34982	0.005	0.019	8.1E-01	34816	0.043	0.020	3.5E-02	34816
rs12671937	7:92654365_G_A	7	92654365	A	G	0.5634	0.016	0.003	9.15E-10	A	G	0.535	0.005	0.013	7.11E-01	69798	-0.001	0.014	9.16E-01	69798	0.014	0.019	4.5E-01	34982	0.001	0.020	9.4E-01	34982	-0.004	0.019	8.2E-01	34816	-0.003	0.020	8.6E-01	34816
rs113520408	7:128402782_G_A	7	128402782	A	G	0.2668	0.017	0.003	1.97E-08	A	G	0.272	0.038	0.015	9.41E-03	69798	-0.005	0.016	7.73E-01	69798	0.036	0.021	7.7E-02	34982	-0.006	0.022	7.8E-01	34982	0.039	0.021	5.7E-02	34816	-0.003	0.022	8.8E-01	34816
rs17167170	7:133302345_A_G	7	133302345	A	G	0.7873	0.02	0.003	1.14E-09	G	A	0.203	-0.080	0.016	9.38E-07	69798	0.011	0.018	5.20E-01	69798	-0.076	0.023	9.2E-04	34982	0.012	0.025	6.3E-01	34982	-0.084	0.023	3.0E-04	34816	0.010	0.025	6.9E-01	34816
rs11768238	7:135227513_G_A	7	135227513	A	G	0.3246	-0.017	0.003	1.00E-09	A	G	0.327	-0.039	0.014	5.14E-03	69798	-0.007	0.015	6.49E-01	69798	-0.035	0.020	8.2E-02	34982	0.040	0.021	5.5E-02	34982	-0.044	0.020	2.6E-02	34816	-0.054	0.021	1.2E-02	34816
rs12682297	8:145712860_T_A	8	145712860	A	T	0.4496	-0.016	0.003	3.93E-09	A	T	0.460	-0.054	0.013	4.62E-05	69798	0.004	0.014	7.72E-01	69798	-0.063	0.019	7.8E-04	34982	0.004	0.020	8.5E-01	34982	-0.045	0.019	1.5E-02	34816	0.004	0.020	8.4E-01	34816
rs1871109	9:1746016_T_G	9	1746016	T	G	0.5616	-0.016	0.003	4.35E-10	G	T	0.444	0.034	0.013	1.09E-02	69798	0.002	0.014	8.76E-01	69798	0.039	0.019	3.6E-02	34982	0.012	0.020	5.3E-01	34982	0.029	0.019	1.2E-01	34816	-0.009	0.020	6.7E-01	34816
rs13294439	9:23358875_A_C	9	23358875	A	C	0.5765	-0.023	0.003	2.20E-17	C	A	0.410	0.081	0.013	1.67E-09	69798	-0.050	0.014	4.79E-04	69798	0.047	0.019	1.3E-02	34982	-0.050	0.020	1.4E-02	34982	0.114	0.019	1.7E-09	34816	-0.051	0.020	1.2E-02	34816
rs895606	9:88003668_A_G	9	88003668	A	G	0.4328	0.015	0.003	2.25E-08	G	A	0.523	-0.020	0.013	1.39E-01	69798	-0.006	0.014	6.60E-01	69798	-0.037	0.019	4.6E-02	34982	0.003	0.020	8.8E-01	34982	-0.002	0.019	9.1E-01	34816	-0.016	0.020	4.3E-01	34816
rs7854982	9:124644562_T_C	9	124644562	T	C	0.4869	-0.015	0.003	1.29E-08	C	T	0.514	0.024	0.013	6.63E-02	69798	0.008	0.014	5.74E-01	69798	0.014	0.019	4.7E-01	34982	0.004	0.020	8.4E-01	34982	0.035	0.019	6.3E-02	34816	0.012	0.020	5.6E-01	34816
rs11191193	10:103802408_A_G	10	103802408	A	G	0.6511	0.018	0.003	5.44E-11	G	A	0.342	-0.056	0.014	5.61E-05	69798	0.008	0.015	5.81E-01	69798	-0.063	0.019	1.2E-03	34982	0.008	0.021	7.0E-01	34982	-0.049	0.020	1.3E-02	34816	0.008	0.021	7.1E-01	34816
rs12772375	10:104082688_T_G	10	104082688	T	G	0.416	-0.015	0.003	1.56E-08	G	T	0.581	0.044	0.013	1.16E-03	69798	-0.015	0.014	3.01E-01	69798	0.042	0.019	2.8E-02	34982	-0.004	0.020	8.3E-01	34982	0.045	0.019	1.8E-02	34816	-0.026	0.021	2.2E-01	34816
rs7945718	11:12748819_A_G	11	12748819	A	G	0.5951	0.015	0.003	1.54E-08	G	A	0.411	-0.035	0.013	8.23E-03	69798	-0.003	0.014	8.18E-01	69798	-0.032	0.019	9.0E-02	34982	-0.013	0.020	5.2E-01	34982	-0.039	0.019	4.1E-02	34816	0.006	0.021	7.7E-01	34816
rs7955289	12:14653667_T_A	12	14653667	A	T	0.6213	0.017	0.003	4.49E-10	A	T	0.612	0.025	0.013	5.97E-02	69798	0.006	0.014	6.92E-01	69798	0.053	0.019	5.6E-03	34982	0.019	0.020	3.6E-01	34982	-0.001	0.019	9.4E-01	34816	-0.007	0.021	7.2E-01	34816
rs2456973	rs2456973	12	56416928	A	C	0.6791	-0.02	0.003	1.06E-12	C	A	0.334	0.046	0.014	1.00E-03	69798	-0.024	0.015	1.06E-01	69798	0.025	0.020	1.9E-01	34982	-0.024	0.021	2.5E-01	34982	0.066	0.020	7.9E-04	34816	-0.023	0.021	2.8E-01	34816
rs7131944	12:92159557_A_T	12	92159557	A	T	0.7164	0.015	0.003	9.00E-09	T	A	0.372	-0.007	0.014	6.02E-01	69798	0.019	0.015	1.86E-01	69798	0.012	0.019	5.3E-01	34982	0.025	0.021	2.3E-01	34982	-0.026	0.019	1.7E-01	34816	0.014	0.021	5.1E-01	34816
rs572016	12:121279083_G_A	12	121279083	A	G	0.4907	0.014	0.003	3.46E-08	A	G	0.491	-0.027	0.013	3.72E-02	69798	-0.015	0.014	2.99E-01	69798	-0.016	0.019	3.7E-01	34982	0.002	0.020	9.4E-01	34982	-0.038	0.019	4.3E-02	34816	-0.031	0.020	1.3E-01	34816
rs7306755	12:123767929_G_A	12	123767929	A	G	0.2257	0.023	0.003	1.26E-12	A	G	0.200	0.069	0.016	1.99E-05	69798	-0.035	0.017	4.25E-02	69798	0.050	0.023	3.0E-02	34982	-0.022	0.024	3.6E-01	34982	0.090	0.023	9.4E-05	34816	-0.049	0.025	5.1E-02	34816
rs9537821	13:58402771_A_G	13	58402771	A	G	0.7631	0.024	0.003	1.50E-16	G	A	0.268	-0.069	0.015	2.78E-06	69798	-0.016	0.016	3.10E-01	69798	-0.067	0.021	1.2E-03	34982	-0.036	0.022	1.0E-01	34982	-0.070	0.021	7.1E-04	34816	0.004	0.022	8.4E-01	34816
rs1043209	14:23373986_G_A	14	23373986	A	G	0.6026	0.018	0.003	1.82E-11	A	G	0.604	0.021	0.013	1.26E-01	69798	0.014	0.014	3.37E-01	69798	0.015	0.019	4.4E-01	34982	0.031	0.020	1.3E-01	34982	0.026	0.019	1.7E-01	34816	-0.003	0.021	8.8E-01	34816
rs17119973	14:84913111_G_A	14	84913111	A	G	0.3078	-0.019	0.003	3.55E-10	A	G	0.256	-0.055	0.015	2.24E-04	69798	0.002	0.016	9.22E-01	69798	-0.062	0.021	3.4E-03	34982	-0.011	0.023	6.3E-01	34982	-0.049	0.021	2.2E-02	34816	0.015	0.023	5.3E-01	34816
rs12969294	18:35186122_A_G	18	35186122	A	G	0.3787	-0.016	0.003	7.24E-09	G	A	0.659	0.064	0.014	4.09E-06	69798	-0.038	0.015	1.15E-02	69798	0.068	0.020	5.2E-04	34982	-0.047	0.021	2.4E-02	34982	0.060	0.020	2.3E-03	34816	-0.029	0.021	1.8E-01	34816
rs2837992	21:42620520_T_C	21	42620520	T	C	0.3881	0.015	0.003	3.80E-08	C	T	0.633	-0.038	0.014	5.27E-03	69798	-0.003	0.015	8.39E-01	69798	-0.022	0.019	2.5E-01	34982	-0.007	0.020	7.2E-01	34982	-0.053	0.019	5.6E-03	34816	0.001	0.021	9.5E-01	34816
rs165633	22:29880773_A_G	22	29880773	A	G	0.7948	-0.018	0.003	2.86E-09	G	A	0.230	0.006	0.016	7.09E-01	69798	-0.008	0.017	6.36E-01	69798	0.009	0.022	6.8E-01	34982	-0.019	0.024	4.4E-01	34982	0.002	0.022	9.1E-01	34816	0.002	0.024	9.5E-01	34816
